# Screening and identification of miRNAs regulating *Tbx4/5* genes of *Pampus argenteus*

**DOI:** 10.7717/peerj.14300

**Published:** 2022-10-24

**Authors:** Cheng Zhang, Shun Zhang, Mengdi Liu, Yajun Wang, Danli Wang, Shanliang Xu

**Affiliations:** 1 Ningbo University, Zhejiang, China; 2 Key Laboratory of Applied Marine Biotechnology, Ningbo University, Ningbo, China

**Keywords:** *Pampus argenteus*, Pelvic fins, MicroRNA, Tbx4/5, Development

## Abstract

**Background:**

Silver pomfret (*Pampus argenteus*) is one of the most widely distributed and economically important pelagic fish species. However, an unique morphological feature of *P. argenteus* is the loss of pelvic fins, which can increase the energy requirement during food capture to some extent and is therefore not conducive to artificial culture. *Tbx4/5* genes are highly conserved regulatory factors that regulate limb development in vertebrates and are in turn regulated by microRNAs (miRNAs). However, the miRNAs that directly regulate the *Tbx4/5* genes in *P. argenteus* remain to be elucidated.

**Methods:**

The *Tbx4/5* genes of *P. argenteus* were first cloned, and the small RNA transcriptomes were sequenced by high-throughput sequencing during the critical period of the fin development at days 1, 7, and 13 of hatching. The miRNAs regulating the *Tbx4/5* genes of *P. argenteus* were subsequently predicted by bioinformatics analysis, and the related miRNAs were verified *in vitro* using a dual fluorescence reporter system.

**Results:**

A total of 662 miRNAs were identified, of which 257 were known miRNAs and 405 were novel miRNAs were identified. Compared to day 1, 182 miRNAs were differentially expressed (DE) on day 7, of which 77 and 105 miRNAs were downregulated and upregulated, respectively, while 278 miRNAs were DE on day 13, of which 136 and 142 miRNAs were downregulated and upregulated, respectively. Compared to day 13, four miRNAs were DE on day 7, of which three miRNAs were downregulated and one miRNA was upregulated. The results of hierarchical clustering of the miRNAs revealed that the DE genes were inversely expressed between days 1 and 7, and between days 1 and 13 of larval development, indicating that the larvae were in the peak stage of differentiation. However, the number of DE genes between days 7 and 13 of larval development was relatively small, suggesting the initiation of development. The potential target genes of the DE miRNAs were subsequently predicted, and Gene Ontology (GO) and Kyoto Encyclopedia of Genes and Genomes (KEGG) enrichment analyses of target genes were performed. The results suggested that the DE miRNAs were involved in growth, development, and signal transduction pathways, of which the Wnt and Fgfs signaling pathways are known to play important roles in the growth and development of fins. The results of dual fluorescence reporter assays demonstrated that miR-102, miR-301c, and miR-589 had a significant negative regulatory effect on the 3′-UTR of the *Tbx4* gene, while miR-187, miR-201, miR-219, and miR-460 had a significant negative regulatory effect on the 3′-UTR of the *Tbx5* gene. Altogether, the findings indicated that miRNAs play an important role in regulating the growth and development of pelvic fins in *P. argenteus*. This study provides a reference for elucidating the interactions between the miRNAs and target genes of *P. argenteus* in future studies.

## Introduction

The expression of T-box genes follows a complex spatio-temporal pattern during development, and the genes encode transcription factors that binds to DNA in a sequence-specific manner (Papaioannou & Silver, 1998; Papaioannou, 2001). The sequences of the encoded proteins contain a highly conserved DNA binding domain. The *Tbx2/3/4/5* gene subfamily in the T-box family plays important roles in the development of vertebrate appendages, heart, and eyes (Horton et al., 2008). Of these, the *Tbx4*/*5* genes are highly conserved regulatory factors of limb development in vertebrates. For instance, *Tbx4* knockout in zebrafish (*Danio rerio*) results in the complete loss of pelvic fins without altering the other morphological characteristics. These findings indicate that the loss of the *Tbx4* gene is the key reason underlying the loss of pelvic fins in zebrafish (Lin et al., 2016). Ahn et al. (2002) observed that the morpholine-induced knockout of *Tbx5* in zebrafish inhibited the migration of mesodermal cells from the lateral plate to the site of growth of fin buds, which resulted in the loss of pectoral fins. A study on ecological limb induction in chickens also demonstrated that the expression of *Tbx4*/*5* genes is related to limb development. *Tbx4* stimulates the formation of appendage buds and induces the formation of leg-like structures, while *Tbx5* promotes the formation of wing-like appendages, indicating that *Tbx4*/*5* might play key roles in limb induction (Naiche & Papaioannou, 2003; Takeuchi et al., 2003; Naiche & Papaioannou, 2007). Similar mechanisms have been reported in other species, including *Notophthalmus viridescens* and *Anguilla japonica*, in which *Tbx4* and *Tbx5* were reported to be involved in the formation of hind limbs and forelimbs, respectively (Simon et al., 1997; Khan, Linkhart & Simon, 2002; Chen et al., 2019). Silver pomfret (*Pampus argenteus*) is an economically important fish species, and its nutritive value and medicinal applications are widely known (Xu et al., 2012; Zhang et al., 2014). *P. argenteus* is a highly marketed fish species in the aquaculture industries of Kuwait, India and China; however, the phenomenon of “pelvic fins-loss” has also been observed in *P. argenteus*. *P. argenteus* is known to consume jellyfishes in the natural habitat (Tong, Li & Guo, 2013); however, *P. argenteus* often mistakes bubbles on the water surface for jellyfish during artificial breeding. After consuming large quantities of bubbles, the fishes may find it difficult to maintain balance. Fins serve as appendages and are important organs for movement and maintaining balance (Su, 2014). The lack of pelvic fins in *P. argenteus* could be one of the reasons underlying the tendency to roll over, which may increase the energy requirement while capturing the same quantity of food, thus indirectly increasing the difficulty of artificially breeding *P. argenteus*.

*Tbx4*/*5* genes play a highly conserved role in the forelimb and hindlimb determination in vertebrates, and are regulated by miRNAs. MiRNAs are single-stranded endogenous non-coding RNAs (ncRNAs) that pair with the complementary regions of the 3′-UTR of target mRNAs to form RNA-induced silencing complexes, thus regulating the expression of corresponding functional proteins. MiRNAs participate in various physiological reactions by regulating the post-transcriptional expression of target genes and participating in various physiological processes (Krol, Loedige & Filipowicz, 2010; Meijer et al., 2013). For instance, Yin et al. (2008) observed the dynamic regulation of several miRNAs during the process of fin regeneration in zebrafish. In particular, the study demonstrated that the expression level of miR-133 is lower in injured fins but high during regeneration, which indicated that miR-133 could be involved in regulating the *Tbx5* gene. Similarly, D’Aurizio et al. (2016) reported that miR-30, miR-34, miR-190, and miR-21 regulate the expression levels of *Tbx5* in zebrafish. Additionally, Chiavacci et al. (2012) observed that the downregulation of miR-218 in zebrafish embryos restores the cardiac defects caused by *Tbx5* overexpression. Previous studies have demonstrated that miRNAs play unique roles in different tissues of fishes, including the fins (miR-133) (Yin et al., 2008), bones (miR-140, miR-204, miR-211) (Huang et al., 2010; Nakamura et al., 2012), eyes (miR-184, let-7, miR-183-1) (Ramachandran, Fausett & Goldman, 2010; Xia et al., 2011), muscles (miR-199, miR-203b) (Yan et al., 2013; de Paula et al., 2017), and heart (miR-128, miR-138, miR-218, miR-21, miR-142a-3p) (Morton et al., 2008; Fish et al., 2011; Chiavacci et al., 2012; Lalwani et al., 2012; Banjo et al., 2013). These findings indicated that miRNAs partake in regulating the growth and development of various fishes. To date, however, there is a scarcity of relevant reports on whether the *Tbx4/5* genes of *P. argenteus* are regulated by miRNAs. The only study by Zhu (2020) speculated that the *Tbx4/5* genes of *P. argenteus* might be regulated by certain miRNAs.

Our previous study demonstrated that the expression level of *Tbx4* mRNA peaked on day 13 of the larval period of *P. argenteus*, and the positive signal of RNA hybridization was detected in the abdominal epithelium; however, the expression of *Tbx4* protein was significantly inhibited. Of all the tissues of adult *P. argenteus*, the protein expression of *Tbx4* protein was lowest in the abdominal epithelium, which indicated that the expression of *Tbx4* protein was also inhibited in the abdominal epithelium of adult *P. argenteus*. Morphological examination revealed that the pectoral fins appeared on day 1, the caudal fin cells appeared on day 7, while the dorsal and gluteal fin cells began accumulating on day 13. The fins of juvenile fishes developed completely at day 19, without the development of pelvic fins and obvious cell accumulation (Zhu, 2020). We speculated that the translation of the protein encoded by the *Tbx4* gene was inhibited by other factors during the critical development of pelvic fins in *P. argenteus* (days 7–13 of larval development), which inhibited the formation and differentiation of pelvic fins. In this study, we therefore selected these three stages (days 1, 7, and 13) of pelvic fin development in *P. argenteus* and constructed the corresponding small RNA (sRNA) transcriptomes. The miRNAs that regulate the *Tbx4/5* genes of *P. argenteus* were subsequently screened and identified by bioinformatics analyses, and the results were verified *in vitro* using a dual fluorescence reporter system. Altogether, this study aimed to elucidate the molecular regulatory mechanism underlying the loss of pelvic fins in *P. argenteus*.

## Methods

### Sample preparation, sRNA isolation, and cDNA library construction

The samples of *P. argenteus* used for the experiments were farmed from May to July 2021 in Xixuan Island Breeding Base, Zhejiang Marine Fisheries Research Institute, Zhoushan, Zhejiang Province (28°31′56′′ N, 122°12′24′′ E). The fishes were cultured in indoor cement pools of 25 m^2^ area, and a water depth of 1.4 m. The transcriptomes of *P. argenteus* were sequenced at days 1, 7, and 13 of larval growth, and two biological replicates were sequenced for each stage. The data represented the mean of two biological replicates for the three individual transcriptomes, and were labelled O_D (A, B), S_D (A, B), and T_D (A, B). The total RNA was extracted in 1.5 ml centrifuge tubes (Guangzhou Jet Bio-Filtration Co., Ltd., Guangzhou, China) by the Trizol method. RNA degradation and contamination were monitored using 1% agarose gels, and the purity, concentration, and integrity of the extracted RNA were evaluated. For each sample, 3 μg of total RNA was used as the input material for constructing the sRNA library. The sRNA library was prepared and sequenced using an Illumina HiSeq 2000 NGS platform. The quality of the library was finally evaluated using DNA High Sensitivity Chips on an Agilent Bioanalyzer 2100 system. The protocol for animal experiments was approved by the Animal Health and Use Committee of Ningbo University (permit number: NO20210706).

### Sequence data analyses and qRT-PCR analysis of differentially expressed (DE) miRNAs

The clean reads were obtained by processing the raw reads in FASTQ format using customized Perl and Python scripts. Then, clean reads of certain range of lengths were selected for subsequent analyses. The sRNA tags were mapped to the reference sequence (*P. argenteus* genomic sequence, GenBank ID: JHEK00000000.1) using Bowtie without any mismatches, and their expression and distribution were analyzed (Langmead et al., 2009). The Rfam10.1 and RepeatMasker databases were used for analyzing the snRNAs, snoRNAs, tRNAs, rRNAs, and repetitive sequences. The miRDeep2 software was used for identifying the known miRNAs and predicting the novel miRNAs of *P. argenteus* (Friedländer et al., 2012). The dicer cleavage site, secondary structure, and minimum free energy of the miRNAs were determined using the MIREAP software, following which the sRNAs were annotated and summarized.

The transcriptome of the larvae at different developmental stages was compared for identifying the significantly DE miRNAs (DEMs; |log_2_(fold change)| > 1, *P* ≤ 0.05). The TargetScan and MiRanda software were used for analyzing the differences in gene expression among the different development stages. The predicted target genes were subsequently subjected to Gene Ontology (GO) and Kyoto Encyclopedia of Genes and Genomes (KEGG) enrichment analyses (Mao et al., 2005). In order to verify the sequencing data, eight significantly DE miRNAs were analyzed by qRT-PCR, and the primers used for the specific miRNAs are enlisted in Table 1. U6 was used as the internal control. The qRT-PCR experiments were performed in triplicate, at least three times for each miRNA. The alterations in miRNA expression were determined using the 2^−ΔΔCt^ method.

**Table 1:** Primers used for qPCR of the miRNAs.

miRNA	Primer (5′ to 3′)
dre-miR-19b-5p	AGTTTTGCTGGTTTGCATTCAG
dre-miR-301b-5p	GCTTTGACGATGTTGCACTAC
novel_113	TGATATGTTTGATATTCGGTTGT
novel_589	AAATCTCAGCTGGCAACTGTG
dre-miR-219-3p	GGAGTTGTGGATGGACATCACGC
dre-miR-460-5p	CCTGCATTGTACACACTGTGCG
novel_212	ATCAGTGACATCTTTGGACCGTT
novel_224	TTTACAGGCTATGCTAATCTGT
U6-F	CTCGCTTCGGCAGCACA
U6-R	AACGCTTCACGAATTTGCGT

### Screening of miRNAs that regulate *Tbx4/5* genes, and cloning of *Tbx4/5*

The miRNAs that regulate *Tbx4/5* gene expression were screened using the PITA and miRanda software. A Wayne diagram was constructed using the screening results, and the miRNAs in the intersection area were further selected for identifying the miRNAs that regulate the expression of *Tbx4/5* genes. The total RNA was extracted by the Trizol method, and subsequently reverse transcribed Primers containing the *NotI* and *XhoI* restriction sites, and the GAAT and CCG protective bases, were designed based on the obtained full-length sequence of the *Tbx4/5* genes of *P. argenteus*, and the 3*’*-UTR fragments of the *Tbx4/5* genes were cloned. Site-directed mutagenesis was performed at the corresponding positions based on the 3′-UTR fragments and corresponding miRNA binding sites, using a Mut Express MultiS Fast Mutagenesis Kit V2 (Vazyme Biotech Co., Ltd, Nanjing, China). The primers used for site-directed mutagenesis were designed using the miRNA Design software, and the primers included 4Mut-102n-F/R, 4Mut-301cd-F/R, 4Mut-19bd-F/R, 4Mut-301bd-F/R, 4Mut-113n-F/R, 5Mut-187d-F/R, 5Mut-201n-F/R, 5Mut-219d-F/R, and 5Mut-460d-F/R (Tables 2 and 3). PCR amplification was subsequently performed, and the target fragment was recovered using a Gel Extraction Kit. The pmirGLO plasmid was digested using the *Nhel* and *Xbal* restriction enzymes, and the target fragment was subsequently recovered. Following ligation, transformation, bacterial selection, and verification of the bacterial liquid by PCR, the positive bacterial liquid was sequenced.

**Table 2:** Primers used for constructing the mutated plasmid of the *Tbx5* gene.

The name of the primers	Sequences	Products
*Tbx5*-3′-UTR-F1-1553	CGGCTCGAGGAGTATCCTCGCTACACCCCCAACC	*Tbx5*-3′-UTR sequence
*Tbx5*-3′-UTR-R2-3371	GAATGCGGCCGCATCTGTCTTTATTTCCATAGAACAG	
*Tbx5*-3′-UTR-F2-1555	CGGCTCGAGGTATCCTCGCTACACCCCCAACCTG	*Tbx5*-3′-UTR sequence
*Tbx5*-3′-UTR-R1-3366	GAATGCGGCCGCTCTTTATTTCCATAGAACAGAAAAT	
psiCHECK-5F	aattctaggcgatcgctcgagCCTAACCGAACCAGAGGACATACC	psiCHECK-2 plasmid polyclone
psiCHECK-5R	attttattgcggccagcggccgcTCCATAGAACAGAAAATGCACTTTTG	
5Mut-187d-F	AACTGgtcagtaTTTGGTTTATGTTTTTGTAATGAAGCA	Binding site mutation
5Mut-187d-R	CCAAAtactgacCAGTTTGCTACTTTTTTGCACAAAT	
5Mut-201n-F	AAATGtgccagaAGCGTCACGCAGCTGTCACT	Binding site mutation
5Mut-201n-R	ACGCTtctggcaCATTTACCCAGAATGCAGCGG	
5Mut-219d-F	AATGatgccagtAGCGTCACGCAGCTGTCACTG	Binding site mutation
5Mut-219d-R	GACGCTactggcatCATTTACCCAGAATGCAGCGG	
5Mut-460d-F	GGTGTTTTAttacgtcTAACATGTGAGAAGGTGCTTTAACAG	Binding site mutation
5Mut-460d-R	AgacgtaaTAAAACACCTTGGCGAAAGATATAA	
5Mut-212n-F	AGCacagttgcCTGTCCTCTCAGTCACCCCCAG	Binding site mutation
5Mut-212n-R	AGGACAGgcaactgtGCTGCGTGACGCTGAGTTG	
5Mut-224n-F	GAAGGAaagcgcttACACTGTTTCCGCTGCATTCTG	Binding site mutation
5Mut-224n-R	GTGTaagcgcttTCCTTCAGCCTGTAGCGTGGT	

**Table 3:** Primers used for constructing the mutated plasmid of the *Tbx4* gene.

The name of the primers	Sequences	Products
Tbx4-3′-UTR-F1841	CGGCTCGAGTCAGCCTATCCCGCACAAGGTA	*Tbx4*-3′-UTR sequence
Tbx4-3′-UTR-F1836	CGGCTCGAGATTTCTCAGCCTATCCCGCACAAGG	
Tbx4-3′-UTR-R3450	GAATGCGGCCGCCACAAATGCATAAGGGATCATCATC	*Tbx4*-3′-UTR sequence
Tbx4-3′-UTR-R3466	GAATGCGGCCGCGAAATAAGGGGCAATGCACAAATGC	
psiCHECK-4F	aattctaggcgatcgctcgagGTCCTATCAGTACCAAGTGGGCC	psiCHECK-2 plasmid polyclone
psiCHECK-4R	attttattgcggccagcggccgcCTTCTCTTTCTCAAAACGTTTATTTATCA	
4Mut-102n-F	GTTGAagcacttcCTTGGCTCCAAACTCACAACTCC	Binding site mutation
4Mut-102n-R	CCAAGgaagtgctTCAACCACAGTATTATGGGCCA	
4Mut-301cd-F	CCCAGaatgctgCCAAAGCCATTTAAATCCATGAA	Binding site mutation
4Mut-301cd-R	TTTGGcagcattCTGGGTAATGATCATAGCTGTATTTCA	
4Mut-19bd-F	CCCTTatgctcgaTGCAAATGTAAAACAGCCTGAGTG	Binding site mutation
4Mut-19bd-R	TTGCAtcgagcatAAGGGTTTTTTCCATGTCAGTCA	
4Mut-301bd-F	GATTcagtttcCTTGGCTCCAAACTCACAACTCC	Binding site mutation
4Mut-301bd-R	GCCAAGgaaactgAATCAACCACAGTATTATGGGCC	
4Mut-113n-F	GTTGGgtgacagAGAATATAGAGAAATTTAGGTTCTTGGCA	Binding site mutation
4Mut-113n-R	ATTCTctgtcacCCAACCTCTGTTAATAAAATGCACTC	
4Mut-589n-F	GCCAAAAactctaaTCTTACACCATGATGAATAAGGTTAAAG	Binding site mutation
4Mut-589n-R	AGAttagagtTTTTGGCACATGCAAATAAGTGG	

### Construction of dual luciferase reporter gene vector and mutant plasmids

The pMD19-T-TBX4/5-3′-UTR and pSicheck-2 plasmids were digested by *Xho*I and *Not*I. and the desired fragments were purified to obtain the 3′-UTR fragments of the *Tbx4/5* genes and the linearized pSicheck-2 plasmid containing the digestion sites. The 3′-UTR fragments of the *Tbx4/5* genes were ligated into the pSicheck-2 vector, and the resulting recombinant reporter vector was denoted as the pSicheck-3′-UTR-WT plasmid. The reporter vector was transformed into DH5α competent cells (TaKaRa, Zhejiang, PR China), and the positive clones were screened in LB medium containing ampicillin. The plasmids were extracted and verified by enzymatic digestion. Using the psiCHECK-3′-UTR-WT plasmid as the template, site-directed mutagenesis was performed at the binding site of the 3′-UTR domains of the corresponding miRNAs. The protocol was performed according to the instructions in the Mut Express MultiS Fast Mutagenesis Kit V2. The mutant plasmids obtained by site-directed mutagenesis were denoted as 3′-UTR-4Mut-102, 3′-UTR-4Mut-301c, 3′-UTR-4Mut-19b, 3′-UTR-4Mut-301b, 3′-UTR-4Mut-113n, 3′-UTR-4Mut-589n; 3′-UTR-5Mut-187d, 3′-UTR-5Mut-201n, 3′-UTR-5Mut-219, and 3′-UTR-5Mut-460d. The plasmids were extracted using Endo Free Plasmid Mini Kit I (Omega Bio-Tek, Norcross, GA, USA). The experimental protocol was performed according to the instructions provided with the kit, and the mutant plasmids were subsequently sequenced.

### Luciferase reporter system

High-confidence DE miRNA-mRNA pairs were selected and targeted for validation. The luciferase activity test confirmed the potential relationship between miRNAs and their target genes. Specifically, the 3′-UTR sequences were synthesized, which contained the miRNA binding sites of the candidate target genes, and subsequently inserted into the multiple cloning sites of the psiCHECK-3′-UTR-WT plasmid. The recombinant psiCHECK-3′-UTR-WT vector and the miRNA mimics or negative control (NC) were co-transfected into DH5α competent cells (TaKaRa, Zhejiang, PR China) at a confluence of 70% using Lipofectamine 3000, according to the manufacturer’s instructions. After 48 h of transfection, the activity of the dual luciferase was detecting using a Dual-Glo^®^ Luciferase Assay Kit (Promega, Madison, WI, USA).

## Results

### Characteristics of the sequencing data

**Figure 1:**
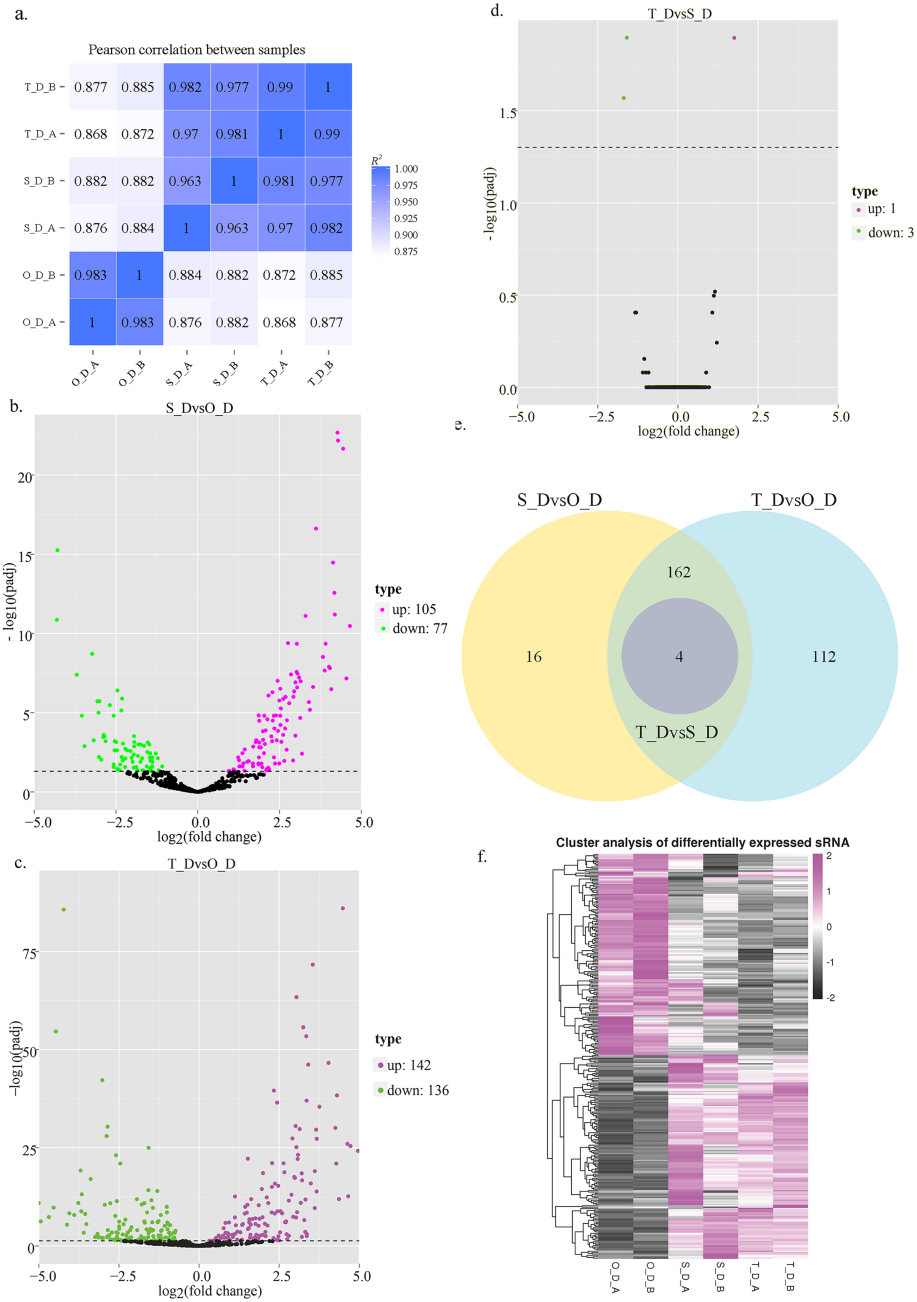
Schematic illustration of miRNA expression in silver pomfret on days 1, 7, and 13. (A) Schematic diagram of the correlation between the miRNA expression levels in the different samples; (B–D) volcano plot of the DE miRNAs in days 1, 7, and 13; (E) venn diagrams of the DE miRNAs among days 1, 7, and 13; (F) hierarchical cluster diagram depicting the miRNA expression levels.

Six sRNA libraries were constructed for the three different developmental stages. The raw transcriptome data of *P. argenteus* were generated using an Illumina sequencing platform, and submitted to the NCBI GenBank database (accession numbers: SAMN20702608–SAMN20702613). The Q20 and Q30 values of all the samples were approximately 98% and 96%, respectively, and the GC content was approximately 48–50%. The length of the 6 sRNA libraries ranged from 11,431,807 to 16,854,046, and the proportion of clean reads to raw reads was above 96%, which indicated that the quality of the sequencing data was highly reliable (Table 4). The lengths of the sRNAs, miRNAs, and siRNAs were 18–35 nt, 21–22 nt, and 24 nt, respectively. Based on the distribution of the sequence lengths of the clean reads, the most abundant size class ranged from 21 to 23 nt (Fig. S1). Additionally, the bias of the first base of known miRNAs was statistically analyzed. The results demonstrated that there was a strong preference for U in the first base of miRNAs in the three different larval stages of *P. argenteus*, and there was an equal proportion of A and C in the first base of novel miRNAs, without any specific preference (Figs. S2 and S3). Figure 1A indicates that the Pearson correlation coefficients among the groups were greater than 0.8, which indicated a strong correlation among the groups, and also implied that the experimental data were highly reliable.

**Table 4:** Filtered transcriptome data of the six sRNA libraries of *P. argenteus*.

Sample ID	Total reads	Raw bases	Clean reads	Q20 (%)	Q30 (%)	GC content (%)
O_D_A	12,890,738	0.645G	12,631,402	98.42	96.35	48.48
O_D_B	13,540,048	0.677G	13,260,379	98.44	96.43	49.30
S_D_A	11,841,376	0.592G	11,431,807	97.41	93.05	49.32
S_D_B	16,082,833	0.804G	15,751,773	98.47	96.52	49.18
T_D_A	17,179,358	0.859G	16,854,064	98.45	96.45	48.52
T_D_B	16,171,454	0.809G	15,826,014	98.43	96.41	49.02

### Identification of DE miRNAs

In this study, we analyzed the expression levels of known and novel miRNAs on days 1, 7, and 13 of the larval stage for determining the significant differences among the 6 sRNA libraries. A total of 662 miRNAs were detected, of which 257 known and 405 novel miRNAs were identified in the 6 sRNA libraries. Compared to day 1, 182 miRNAs were DE on day 7, of which 77 miRNAs were downregulated and 105 miRNAs were upregulated, while 278 miRNAs were DE on day 13, of which 136 miRNAs were downregulated and 142 miRNAs were upregulated. Compared to day 13, 4 miRNAs were DE on day 7, of which 3 miRNAs were downregulated and 1 miRNA was upregulated (Figs. 1B–1E; Table 5). These results indicated that numerous miRNAs are involved in the developmental regulation of *P. argenteus*.

**Table 5:** Comparative analysis of the DE miRNAs among the different treatment groups.

Group	Diff	Up	Down
S_DvsO_D	182	105	77
T_DvsO_D	278	142	136
T_DvsS_D	4	1	3

The results of hierarchical clustering of the miRNAs revealed that the miRNAs had different expression levels among the three different stages of development (Fig. 1F). The genes that were DE between the day 1 (O_D) and day 7 (S_D) larval stages, and between the day 1 (O_D) and day 13 (T_D) larval stages were inversely expressed, indicating that the larvae were in the peak stage of differentiation. However, the number of genes DE between days 7 (S_D) and 13 (T_D) of the larval stages was relatively small, suggesting the initiation of development. Of these, the expression levels of 4 miRNAs (dre-let-7f, dre-miR-129-1-3p, dre-miR-129-3-3p, and novel_154) differed at the three larval developmental periods (Table 6). Compared with those of the O_D larval stage, the expression levels of dre-let-7f, dre-miR-129-1-3p, and dre-miR-129-3-3p miRNAs were upregulated, while those of novel_154 were downregulated in the S_D and T_D larval stages. Compared with those of the T_D larval stages, the expression levels of dre-let-7f were upregulated, while those of dre-miR-129-1-3p, dre-miR-129-3-3p, and novel_154 were downregulated in the O_D and S_D larval stages.

**Table 6:** Descriptive statistics of the DE miRNAs among the different treatment groups.

Group	sRNA	Sample1 readcount	Sample2 readcount	log_2_FoldChange	*P* value	p adj
S_D vs O_D	dre-miR-129-1-3p	448.9748693	14.85402973	4.0854	2.38E−08	3.24E−07
	dre-miR-129-3-3p	448.9748693	14.85402973	4.0854	2.38E−08	3.24E−07
	dre-let-7f	708.9847335	148.4171063	2.1284	3.41E−06	2.59E−05
	novel_154	294.0490602	2,455.690043	−2.547	0.000549	0.0023553
T_D vs O_D	dre-miR-129-1-3p	114.6618467	16.07420681	2.324	0.004672	0.010561
	dre-miR-129-3-3p	114.6618467	16.07420681	2.324	0.004672	0.010561
	dre-let-7f	4,398.063122	162.5455731	4.3633	1.41E−13	1.14E−12
	novel_154	50.10163297	2,666.85176	−5.0053	1.56E−12	1.13E−11
T_D vs S_D	dre-miR-129-1-3p	119.6302503	506.8877433	−1.5932	5.97E−05	0.012706
	dre-miR-129-3-3p	119.6302503	506.8877433	−1.5932	5.97E−05	0.012706
	dre-let-7f	4,595.859641	799.9153518	1.7576	4.26E−05	0.012706
	novel_154	52.25954685	332.6711166	−1.6908	0.000169	0.027027

### Prediction and functional analysis of target genes

**Figure 2:**
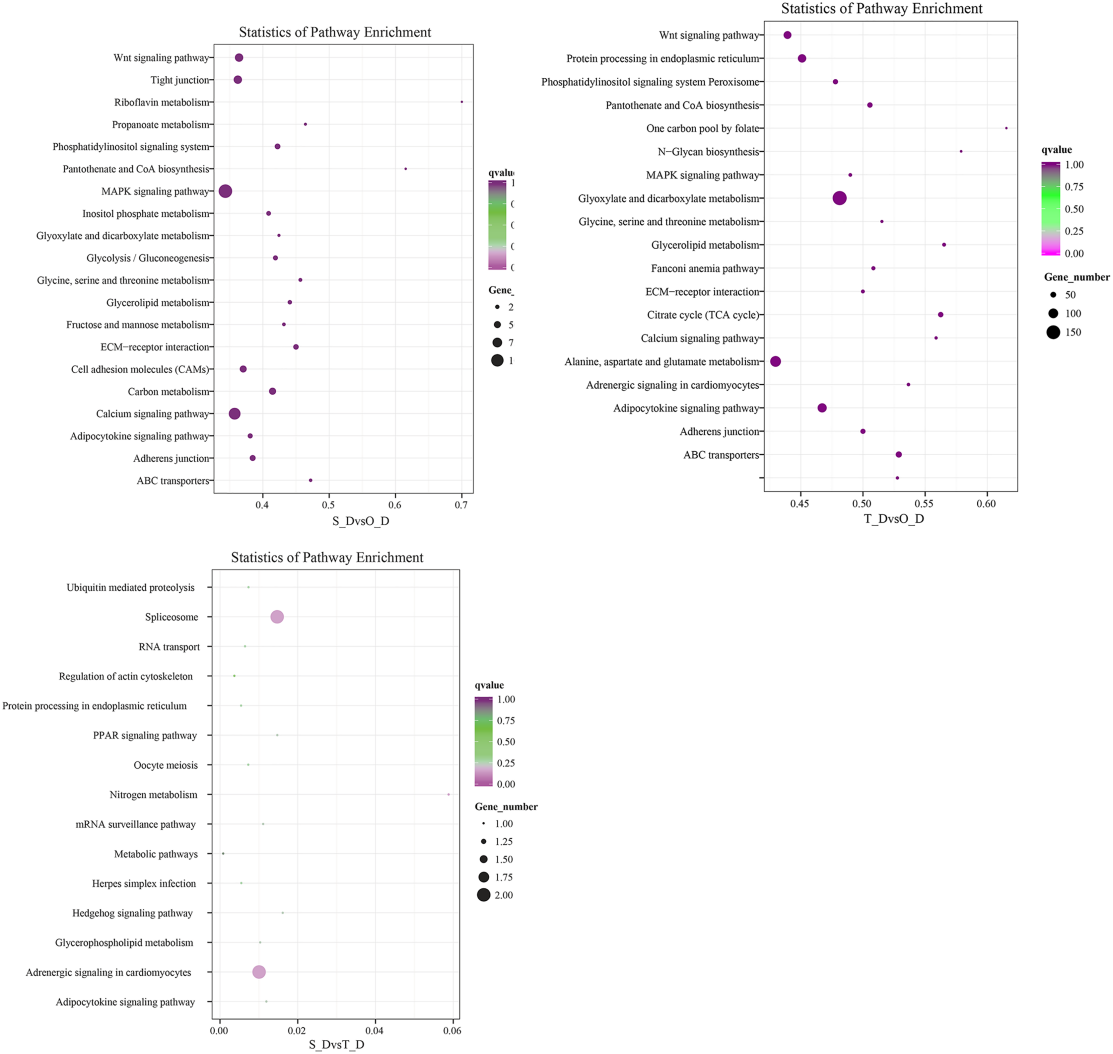
Scatterplot depicting the KEGG pathways of the candidate target genes.

The potential target genes of the DE miRNAs were predicted using the TargetScan webserver. The predicted target genes were classified into three major functional ontologies based on the GO classification system, namely biological process, cellular component, and molecular function. In the biological processes category, the target genes were most enriched in the cellular processes term, followed by the single-cell processes and metabolic processes terms. Additionally, the target genes were most enriched in the cell components category. In the molecular function category, the genes were most enriched in binding and catalytic activity than other terms. The KEGG database (http://www.kegg.jp/kegg/pathway.html) was subsequently used for pathway analysis of the target genes. The 9,440 DE genes in the transcripts of day 1 and day 7 larvae of *P. argenteus* were subjected to pathway enrichment analyses, which revealed that the main enriched pathways were the MAPK signaling pathway, calcium signaling pathway, tight junction, and the Wnt signaling pathway. Pathway enrichment analyses of the 14,236 DE genes in the transcripts of day 1 and day 13 larvae revealed that the main enriched pathways were the MAPK signaling pathway, calcium signaling pathway, Wnt signaling pathway, adrenergic signaling in cardiomyocytes, and protein processing in endoplasmic reticulum. Similarly, the main enriched pathways of the 45 DE genes in the transcripts of day 7 and day 13 larvae were spliceosome and adrenergic signaling in cardiomyocytes (Fig. 2).

### Expression levels of DE miRNAs

**Figure 3:**
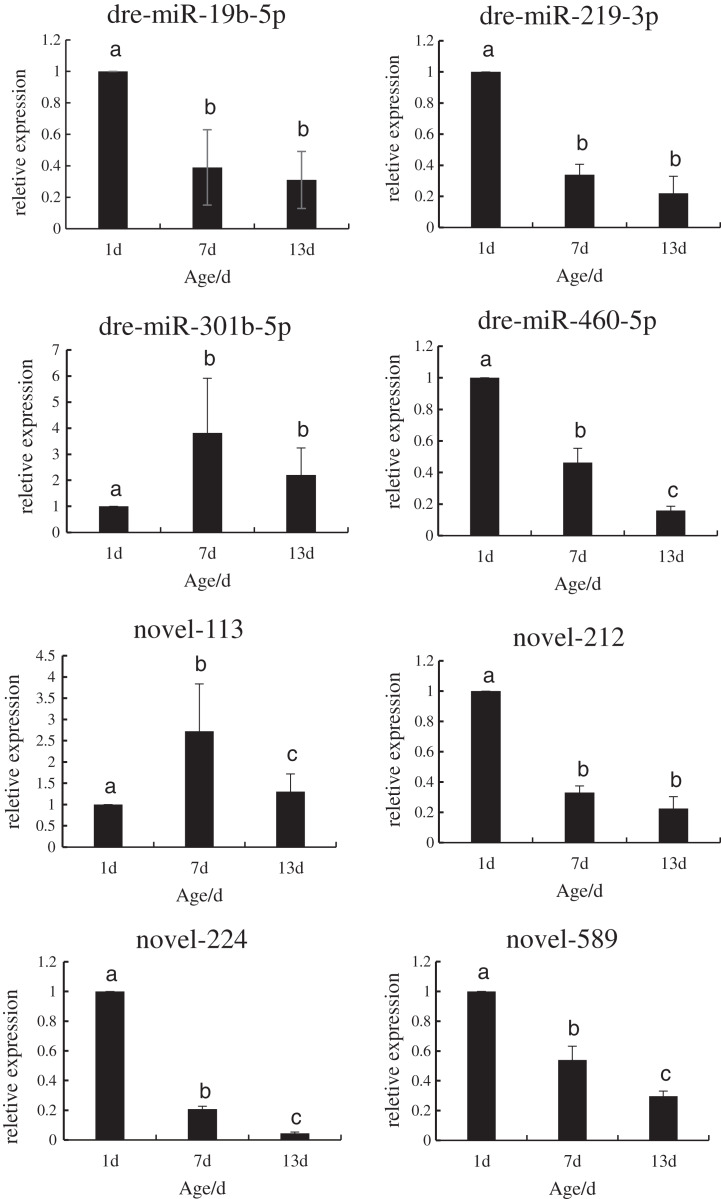
Schematic illustration of the results of RT-qPCR analysis of the eight DE miRNAs.

In order to verify the reliability of the miRNA-Seq data, the expression levels of the DE miRNAs that regulated the target genes during the development of *P. argenteus* were detected by qRT-PCR. As depicted in Fig. 3, the expression levels of dre-miR-19b-5p, dre-miR-219-3p, dre-miR-460-5p, novel-212, novel-224, and novel-589 were downregulated. However, graphical depiction of the expression levels of dre-miR-301b-5p and novel-113 showed an “inverted V” curve, and demonstrated that the expression of these miRNAs peaked on day 7. The expression of these miRNAs was consistent with the patterns determined by high-throughput sequencing. The findings confirmed that the miRNA sequencing data were reliable and could be used in further analyses.

### Screening of miRNAs regulating *Tbx4/5* genes

Similar to the results of previous studies, the expression levels of *Tbx4* mRNA increased continually from day 1 to day 13 in this study, and was highest in day 13 larvae, while the expression of *Tbx5* mRNA showed a downward trend and was highest when the primordia of the 1-day-old pectoral fins appeared. Of the different tissues, the positive signal of the *Tbx4* gene was primarily concentrated in the abdominal epithelium of *P. argenteus*; however, the expression levels of the *Tbx4* protein were low in the abdominal epithelium of day 7 larvae and adult fishes. The positive signal of the *Tbx5* gene was primarily concentrated in the pectoral fins, while the expression of the *Tbx5* protein was relatively high in the pectoral fins of day 1 larvae and adult fishes (Zhu, 2020). We therefore speculated that the *Tbx4/5* genes of *P. argenteus* could be regulated by miRNAs. In this study, we used the miRanda bioinformatics tool (version 2.2a) for analyzing and predicting the miRNAs that regulate the *Tbx4/5* genes of *P. argenteus*. Of these, a total of 6 miRNAs we identified that met the screening criteria. The results of prediction with miRanda revealed that novel_102, dre-miR-301c-3p, dre-miR-19b-5p, dre-miR-301b-5p, novel_113, and novel_589 could regulate the expression of the *Tbx4* gene, while novel_201, dre-miR-187, dre-miR-219-3p, dre-miR-460-5p, novel_212, and novel_224 could regulate the expression of *Tbx5*. The binding sites of the seed sequence of the miRNAs and the 3′-UTR region of the *Tbx4/5* genes were determined, and their distributions are depicted in Figs. S4 and S5 (*Tbx4* GenBank ID: MH709128; *Tbx5* GenBank ID: MH712458).

### Construction of the dual luciferase vector and transfection

**Figure 4:**
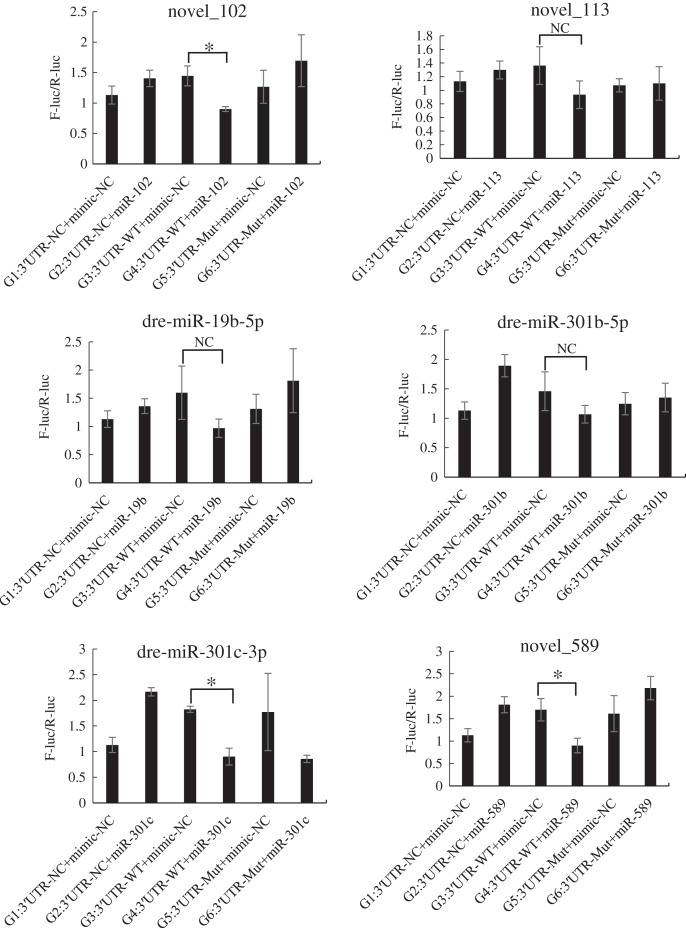
Results of the dual luciferase activity assay of the *Tbx4* gene. An asterisk (*) indicates significant difference among groups.

**Figure 5:**
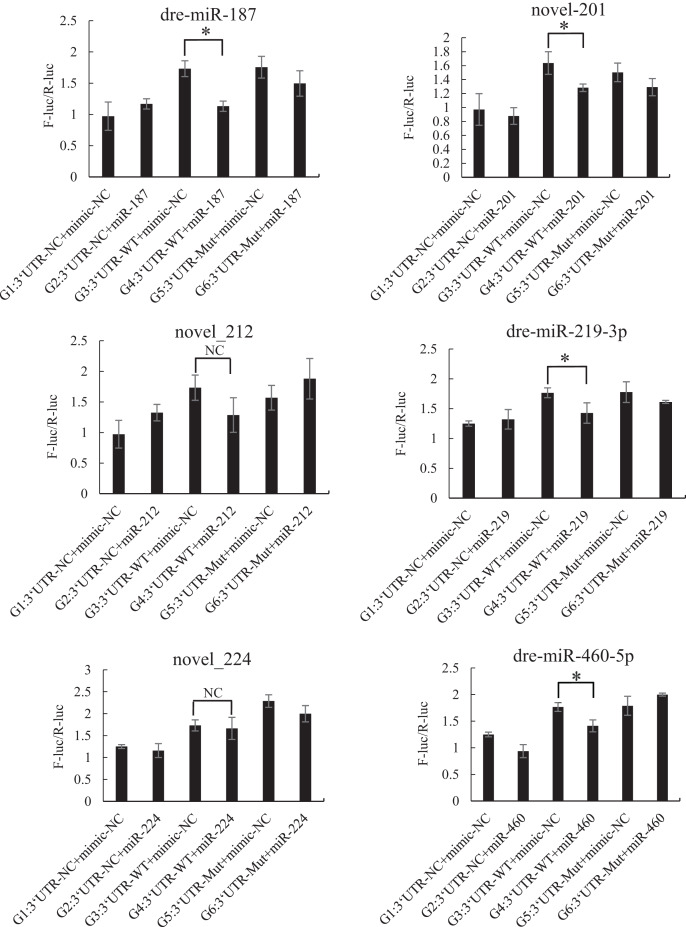
Results of the dual luciferase activity assay of the *Tbx5* gene. An asterisk (*) indicates significant difference among groups.

The mutated recombinant plasmid was successfully constructed using Mut Express MultiS Fast Mutagenesis Kit V2 for site-directed mutagenesis, and the results were verified by sequencing and comparison. The resulting recombinant plasmid was denoted as the pSicheck-3′-UTR-WT plasmid. The sequences of the plasmids obtained by site-directed mutagenesis are depicted in the Figs. S6 and S7. In order to verify the regulation of *Tbx4/5* by the previously identified miRNAs, the psiCHECK-3′-UTR-WT plasmid was co-transfected with the miRNA mimic into HEK293T cells (TaKaRa, Zhejiang, PR China). The cells in the different experimental groups were co-transfected with different plasmids, corresponding to the different predicted regulatory miRNAs, as follows: blank control group (G1): 3′-UTR-NC + miRNA mimic-NC; negative control groups (G2 and G3): (3′-UTR-NC + miRNA mimic-NC) and (3′-UTR-WT + miRNA mimic-NC), respectively; experimental group (G4): 3′-UTR-WT + miRNA mimics (6 miRNA mimics); and point mutation control groups (G5 and G6): (3′-UTR-mut + miRNA mimic-NC and 3′-UTR-mut + miRNA mimic) and (psiCHECK-3′-UTR-mut + miRNA mimic (6 specific miRNA mimics + mimic-NC)). After 48 h of co-transfection, the regulation of *Tbx4/5* by the miRNAs was detected according to the instructions provided in the Dual-Glo®Luciferase Assay System kit, and calculated as the ratio of the activity of the firefly luciferase activity to that of the *Renilla* luciferase, and comparing the ratios calculated for the experimental groups with those of the control group. The results demonstrated that miR-102, miR-301c, and miR-589 had a significant negative regulatory effect on the 3′-UTR of *Tbx4* (*P* < 0.05). The 3′-UTR of *Tbx4* was not significantly regulated by miR-113, miR-19b, and miR-301b; however, the 3′-UTR of *Tbx5* was significantly negatively regulated by miR-187, miR-201, miR-219, and miR-460. The results also demonstrated that the 3′-UTR of *Tbx5* was not significantly regulated by miR-212 and miR-224 (Figs. 4 and 5).

## Discussion

In eukaryotes, miRNAs are primarily involved in the post-transcriptional modification of protein expression. MiRNAs induce post-transcriptional inhibition by pairing with the protein-coding mRNAs, thereby playing a crucial role in gene regulation, and the time- and tissue-specificity of miRNAs have been reported to highly conserved (Bartel, 2009; Krol, Loedige & Filipowicz, 2010). In this study, high-throughput sequencing technologies were used to obtain the whole transcriptome of *P. argenteus* larvae at days 1, 7, and 13 of hatching, and a total of 257 known and 405 novel miRNAs were identified. We observed that some miRNAs were expressed in all the three developmental stages, while some miRNAs were specifically expressed at certain stages. The number of DE miRNAs between day 1 and day 7 larvae, and between day 1 and day 13 larvae was higher than that between day 7 and day 13 larvae. The expression of dre-let-7f, dre-miR-129-1-3p, and dre-miR-129-3-3p miRNAs was upregulated, while that of novel_154 was downregulated in day 7 and day 13 larvae, compared to that of day 1 larvae. The expression of dre-let-7f was upregulated, while that of dre-miR-129-1-3p, dre-miR-129-3-3p, and novel_154 was downregulated in day 13 larvae compared to that of day 7 larvae. Altogether, the results demonstrated that the expression levels of two miRNAs of miR-129 family, namely, dre-miR-129-1-3p and dre-miR-129-3-3p, peaked on the 7^th^ day after hatching, and generally showed an upward trend in all the three larval stages studied herein. MiR-129 is known to have regulatory effects on cell growth, proliferation, and differentiation. Wu et al. (2010) observed that the overexpression of miR-129 in E10 murine lung epithelial cells significantly induces G1 phase arrest, which eventually results in cell death. The study also demonstrated that restoring the expression of the cyclin-dependent kinase 6 gene (*Cdk6*) partially rescued cell growth arrest and cell death phenotype caused by miR-129 overexpression. Altogether, the findings indicated that miR-129 plays an important role in regulating cell proliferation by downregulating the expression of *Cdk6*. We therefore speculated that dre-miR-129-1-3p and dre-miR-129-3-3p may play an important role in cell growth and proliferation in *P. argenteus*. The expression of dre-let-7f was higher and reached a peak on the 13^th^ day after hatching. The let-7 family of miRNAs shows high sequence conservation in animals, and are known to be expressed at any stage of development. This family of miRNAs are organized in clusters or co-expressed with specific coding genes in the genome. Roush & Slack (2008) opined that the expression level of let-7 significantly increases with cellular differentiation in more complex organisms. The results of this study also demonstrated that the expression levels of dre-let-7f also increased steadily in the three larval stages, indicating that the larval cells of *P. argenteus* were in a state of constant differentiation. The expression level of novel_154 continually decreased in the three larval stages, and was lowest on the 13^th^ day after hatching. We speculated that the novel miRNA identified herein (novel_154) and miR-154 may belong to the same family. The expression level of miR-154 was positively correlated with the activation of the Wnt signaling pathway. MiR-154 is known to target the *Dkk2* gene, and potentially inhibits the expression of *Dkk2*. The activity of the classical Wnt signaling pathway is inhibited by *Dkk2*, which negatively regulates osteoclastic activation (Huang, 2017). Altogether, these results indicate that the downregulation of miR-154 inhibits the Wnt signaling pathway, which indirectly leads to the osteoclastic differentiation, maturation, and increased activity. We therefore speculated that novel_154 might negatively regulate the differentiation and proliferation of osteoclasts in the early larval stage of *P. argenteus*, however, the specific functions of novel_154 require further investigation.

The development of appendages is initiated by the formation of lateral buds in the lateral mesoderm (Mariani & Martin, 2003; Freitas, Zhang & Cohn, 2006). The formation of lateral buds is primarily regulated by the T-box transcription factors, *Tbx4*/*5*, and by the fibroblast growth factors, Fgfs8 and Fgfs10, which are expressed in a time-specific manner in the lateral mesoderm for the formation of appendage buds (Qi & Shi, 2016). Downstream of these transcription factors, *Tbx4* activates the Wnt8c/Fgf10 signaling pathway in the lateral plate mesoderm of hindlimbs, while *Tbx5* activates the Wnt2b/Fgf10 signaling pathway in the lateral plate mesoderm of forelimbs, thereby directly inducing limb bud germination (Takeuchi et al., 2003). In the early larval stage of zebrafish, the functional knockout of *Tbx5* leads to the complete loss of pectoral fins (Ahn et al., 2002). Agarwal et al. (2003) observed that *Tbx5* knockout leads to the non-formation of forelimb buds in mice, and inhibition of *Tbx4* and *Tbx5* expression in chicken embryos leads to the non-formation of appendages. It has been additionally demonstrated that the inactivity of Fgf8 during the formation of limb buds results in the formation of very small limb buds (Sun, Mariani & Martin, 2002), while the expression of *Tbx5* regulates the activity of Fgf10 during the formation of pectoral fins in zebrafish and forelimb buds in chicken (Ng et al., 2002). These findings demonstrated that alterations in the expression of *Tbx4/5* genes primarily affect limb bud formation *via* the Wnts and Fgfs signaling pathways. In this study, we observed that the target genes of *P. argenteus* were mostly enriched in the Wnt signaling pathway. We speculated that a certain number of miRNAs may be related to the occurrence, growth, and development of fins in *P. argenteus*, and could activate the Wnt/FGF signaling cascade by regulating expression of *Tbx4*/*5* genes, thereby regulating the development of limb buds.

We subsequently analyzed the miRNAs that regulate the expression of *Tbx4/5* genes, and observed that miR-102, miR-301c, and miR-589 had significant regulatory effects on the 3′-UTR of the *Tbx4* gene. The miR-187, miR-201, miR-219, and miR-224 miRNAs significantly regulated the 3′-UTR of the *Tbx5* gene. These miRNAs have important regulatory effects on gene expression in animals. For instance Pfaff et al. (2011) observed that the miR-301 miRNA family enhances the generation of induced pluripotent stem cells by repressing the homeobox transcription factor, *Meox2*. Lu et al. (2022) reported that the direct targeting of Forkhead box F2 by miR-301b-3p activates the Wnt/β-catenin signaling pathway. Hu et al. (2015) demonstrated that miRNA-589 mediates the epithelial mesenchymal transition of human peritoneal mesothelial cells induced by transforming growth factor (TGF)-β, and epithelial mesenchymal transition is known to be a crucial factor in the development and progression of peritoneal fibrosis. A previous study demonstrated that the Wnt signaling pathway is activated following the inhibition of miR-219-5p (Zhao et al., 2018). These miRNAs also play important roles in regulating gene expression in *P. argenteus*. The expression of *FGF* genes in the lateral mesoderm is induced by the Wnt signaling pathway, leading to limb development (Tanaka et al., 2005). By comparing the results obtained herein with the results of the study by Zhu (2020), we speculated that the expression of *Tbx4* protein in day 7 *P. argenteus* larvae may be regulated by miR-102, miR-301c, and miR-589, and the reduction in *Tbx5* protein expression may be negatively regulated by miR-187, miR-201, miR-219, and miR-224 in day 1 to day 13 larvae.

The process of appendage development is complex and includes three stages, namely, limb location, limb bud initiation, and limb bud growth, and involves the interactions of multiple genes. It has been demonstrated that *Tbx4* and *Tbx5* are involved in the regulation of limb bud initiation (Tanaka, 2005). The *Tbx4* gene is mainly expressed in hindlimbs/pelvic fins, while the *Tbx5* gene is primarily expressed in forelimbs/pectoral fins (Gibson-Brown et al., 1996; Zhang, 2012), indicating that *Tbx4*/*5* can specifically determine limb characteristics. This study confirmed that certain microRNAs regulate the expression of *Tbx4* in *P. argenteus*, which indirectly inhibited the expression of the *Tbx4* protein. This study provides a theoretical basis for further investigations of the relationship between the identified miRNAs and the target genes, *Tbx4* and *Tbx5*. Elucidating the relationship between these miRNAs and target genes will indeed aid in the artificial cultivation of *P. argenteus*. Artificial control of the development of the pelvic fins of *P. argenteus* may aid in improving the balancing ability, thereby reducing the energy requirement for the survival of *P. argenteus* to some extent. This would indirectly reduce the cost of artificial breeding of *P. argenteus*.

In order to elucidate the genetic basis of the “loss of pelvic fins” in *P. argenteus*, subsequent studies should be performed, by microinjection of miRNA mimics or inhibitors into fertilized eggs (or larval stages before fin formation) of *P. argenteus* for overexpressing or suppressing miRNA expression. The expression levels of *Tbx4/5* genes following miRNA silencing or overexpression can be subsequently detected by real-time PCR and western blotting for verifying the regulatory effect of miRNAs on the corresponding target *Tbx4/5* genes. Following the construction of the *Tbx4* gene overexpression plasmid, the *Tbx4* overexpression vector can be injected into fertilized eggs of *P. argenteus*. The existence and transcription of plasmids in the tissues of *P. argenteus* can be subsequently detected, and the morphological changes in *P. argenteus* need to be observed for verifying whether *Tbx4* regulates the loss of pelvic fins in *P. argenteus*.

## Supplemental Information

10.7717/peerj.14300/supp-1 Supplemental Information 1 Raw Data.

10.7717/peerj.14300/supp-2 Supplemental Information 2 Distribution of the sequence lengths of the clean reads.

10.7717/peerj.14300/supp-3 Supplemental Information 3 Schematic diagrams depicting the matching information between sRNAs and miRNAs. The horizontal axis represents the position of the miRNA bases, and the vertical axis represents the percentage of A/U/C/G bases in the miRNAs at the corresponding positions.

10.7717/peerj.14300/supp-4 Supplemental Information 4 Schematic diagrams of the preference of the first base in known miRNAs. The horizontal axis represents the position of the miRNA bases, and the vertical axis represents the percentage of A/U/C/G bases in the miRNAs at the corresponding positions.

10.7717/peerj.14300/supp-5 Supplemental Information 5 Distribution of the binding sites in the seed sequence of the miRNAs and 3ʹ-UTR of the *Tbx4* gene. The double underlines at the beginning and tail regions represent the two restriction sites and protective bases in the 3′-UTR fragment. The labeled residues indicate the binding sites of the miRNA and sequences complementary to the 3′-UTR.

10.7717/peerj.14300/supp-6 Supplemental Information 6 Distribution of the binding sites of the seed sequence of the miRNAs and 3ʹ-UTR of the *Tbx5* gene.. The double underlines at the beginning and tail regions represent the two restriction sites and protective bases in the 3′-UTR fragment. The labeled residues indicate the binding sites of the miRNA and sequences complementary to the 3′-UTR.

10.7717/peerj.14300/supp-7 Supplemental Information 7 Schematic diagram of the *Tbx4* gene following site-directed mutation. The double underlines at the beginning and tail regions represent the two restriction sites and protective bases in the 3′-UTR fragment. The labeled residues indicate the binding sites of the miRNA and sequences complementary to the 3′-UTR.

10.7717/peerj.14300/supp-8 Supplemental Information 8 Schematic diagram of the *Tbx5* gene following site-directed mutation. The double underlines at the beginning and tail regions represent the two restriction sites and protective bases in the 3′-UTR fragment. The labeled residues indicate the binding sites of the miRNA and sequences complementary to the 3′-UTR.

## Additional Information and Declarations

### DNA Deposition

The following information was supplied regarding the deposition of DNA sequences: The raw transcriptome data of *P. argenteus* are available at NCBI GenBank: SAMN20702608–SAMN20702613.

### Data Availability

The following information was supplied regarding data availability: The raw measurements are available in the Supplemental Files.
