# Supplementary material for: Screening and identification of miRNAs regulating Tbx4/5 genes of Pampus argenteus
Source: PeerJ. 2022 Oct 24;10:e14300. doi: 10.7717/peerj.14300 (PMC9610670; doi:10.7717/peerj.14300)
Supplement: Supplemental Information 2 [file peerj-10-14300-s002.pdf]

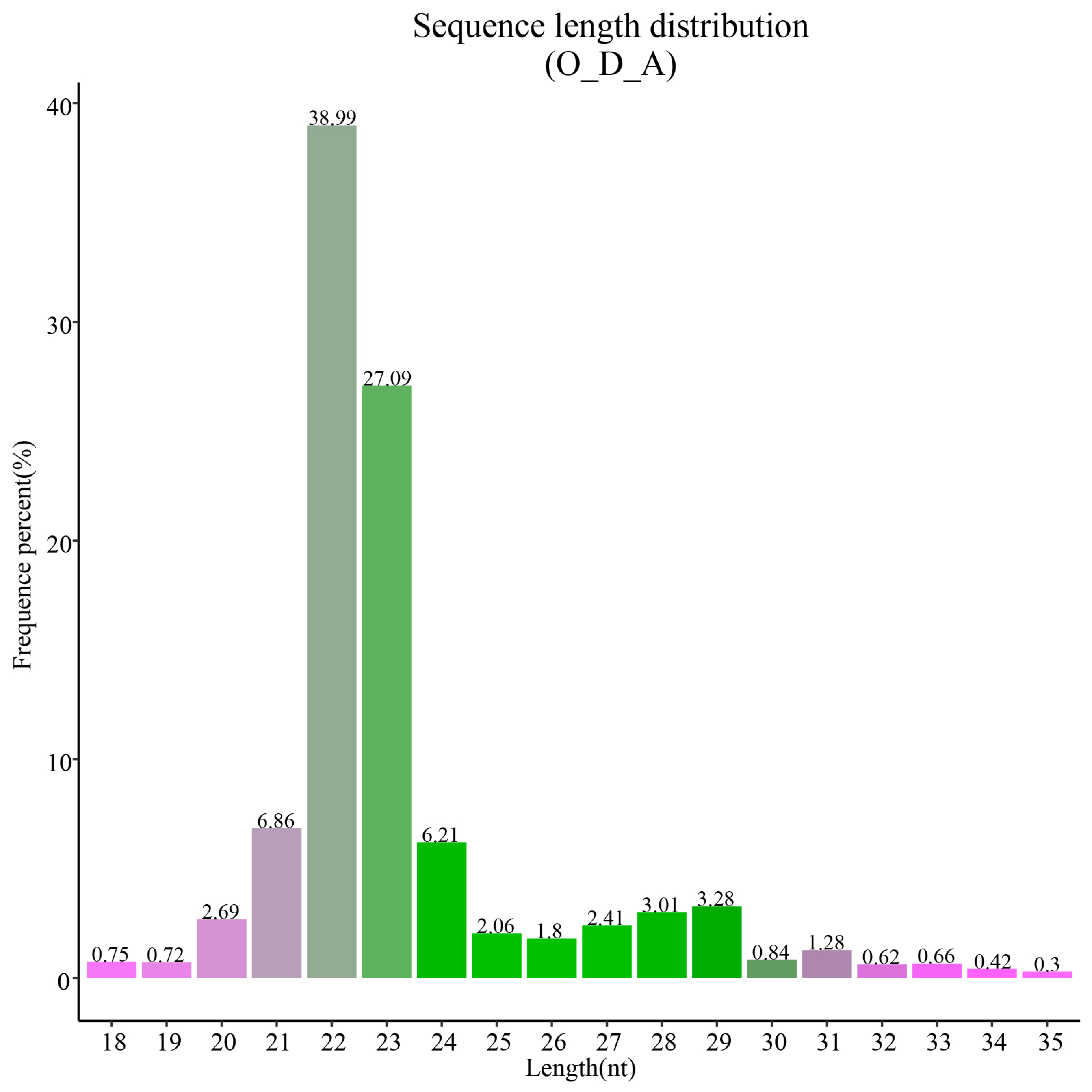

Sequence length distribution
(O_D_A)
Frequence percent(%)
Length(nt)
0
10
20
30
40
0.75
0.72
2.69
6.86
38.99
27.09
6.21
2.06
1.8
2.41
3.01
3.28
0.84
1.28
0.62
0.66
0.42
0.3
18
19
20
21
22
23
24
25
26
27
28
29
30
31
32
33
34
35

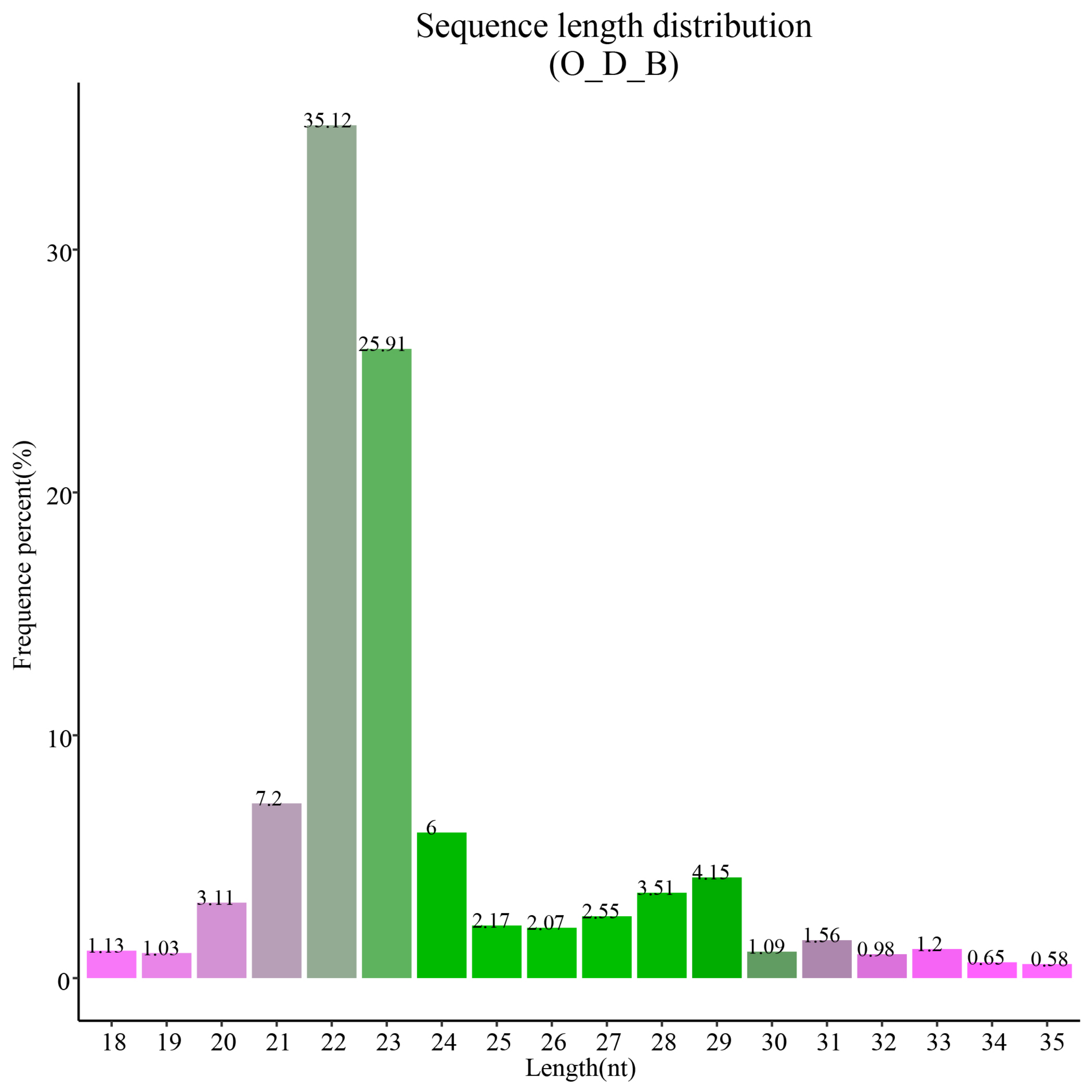

Sequence length distribution
(O_D_B)
Frequence percent(%)
Length(nt)
0
10
20
30
35.12
25.91
7.2
6
4.15
3.51
3.11
2.55
2.17
2.07
1.56
1.2
1.13
1.09
1.03
0.98
0.65
0.58
18
19
20
21
22
23
24
25
26
27
28
29
30
31
32
33
34
35

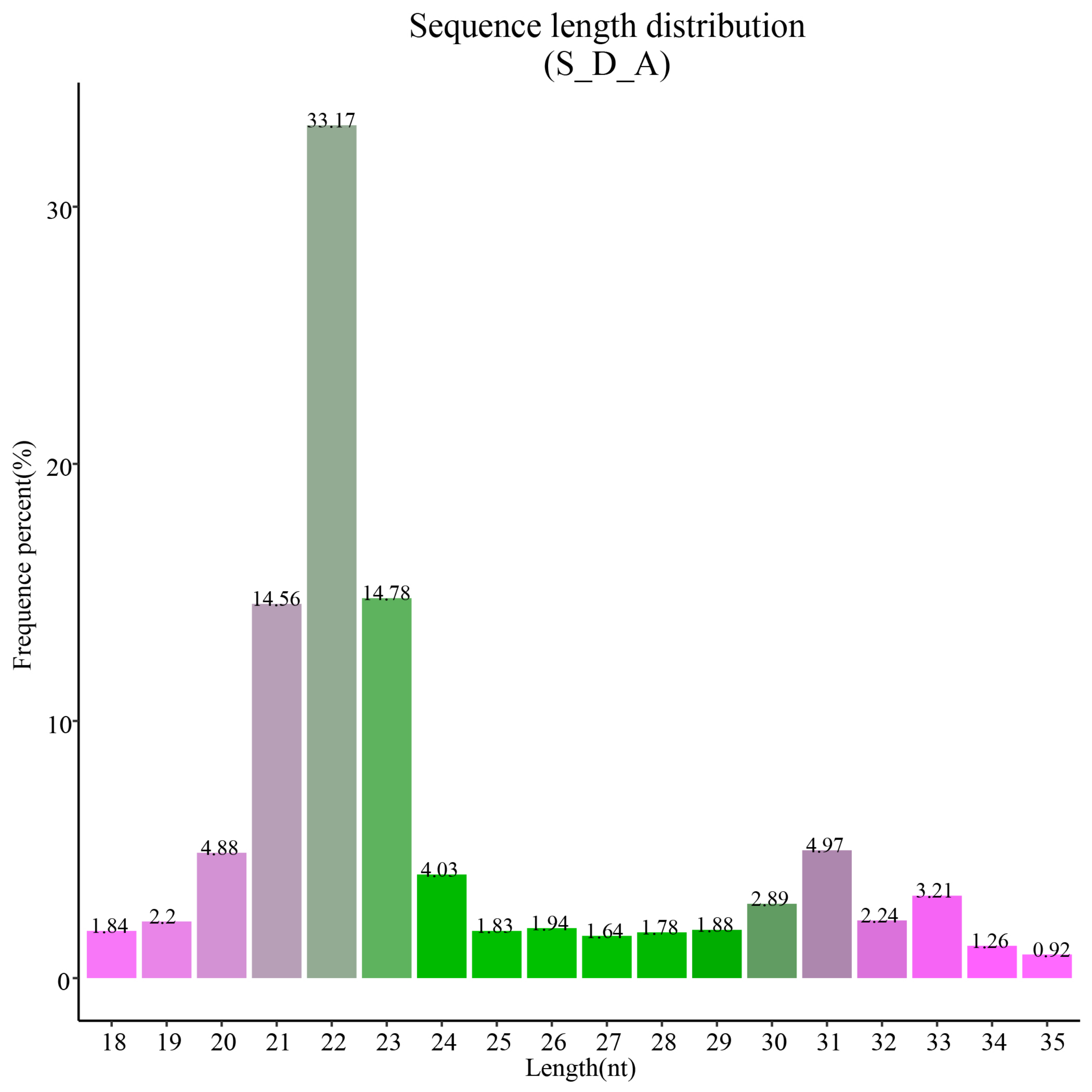

Sequence length distribution
(S_D_A)
Frequence percent(%)
Length(nt)
0
10
20
30
18
19
20
21
22
23
24
25
26
27
28
29
30
31
32
33
34
35
1.84
2.2
4.88
14.56
33.17
14.78
4.03
1.83
1.94
1.64
1.78
1.88
2.89
4.97
2.24
3.21
1.26
0.92

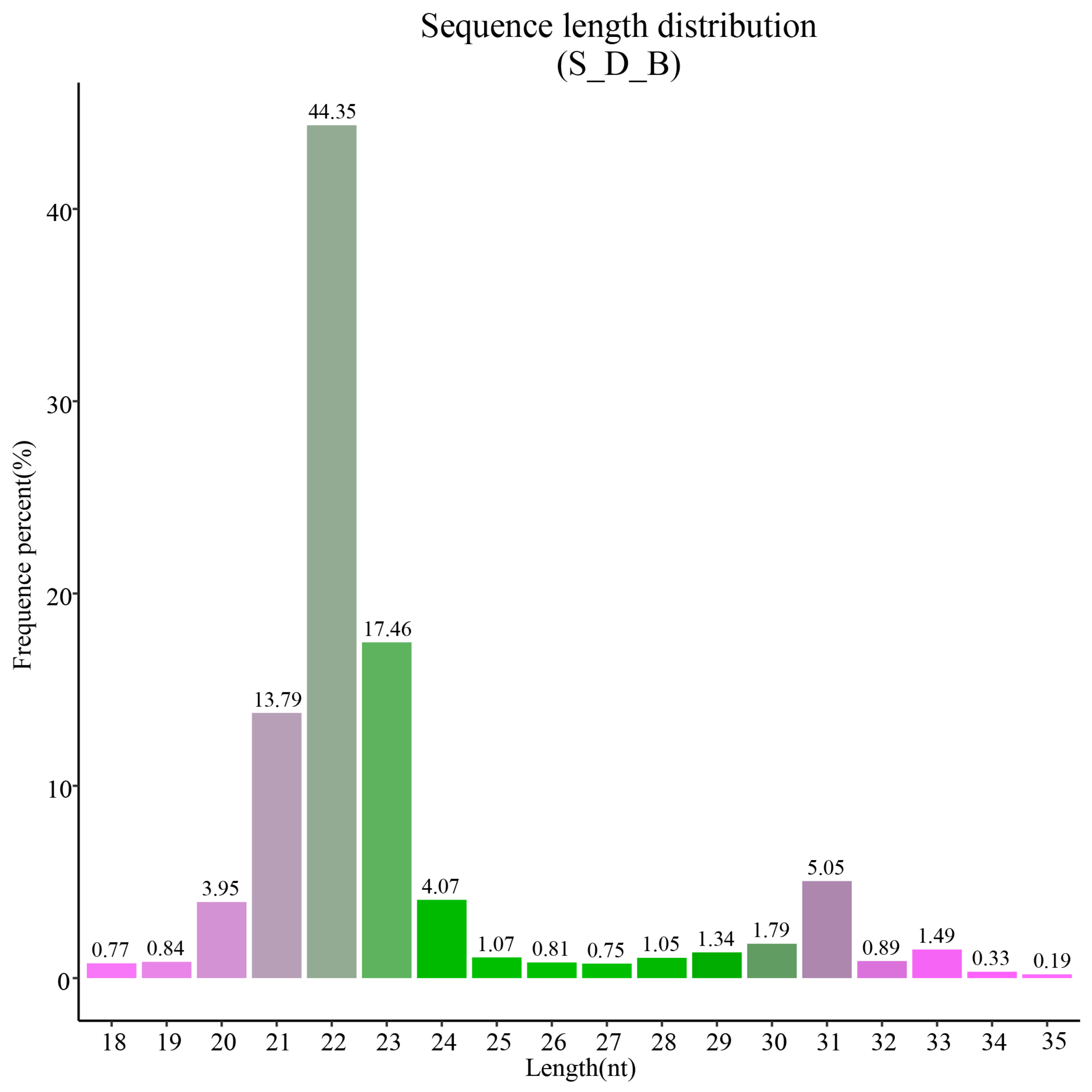

Sequence length distribution
(S_D_B)
Frequence percent(%)
Length(nt)
0
10
20
30
40
0.77
0.84
3.95
13.79
44.35
17.46
4.07
1.07
0.81
0.75
1.05
1.34
1.79
5.05
0.89
1.49
0.33
0.19
18
19
20
21
22
23
24
25
26
27
28
29
30
31
32
33
34
35

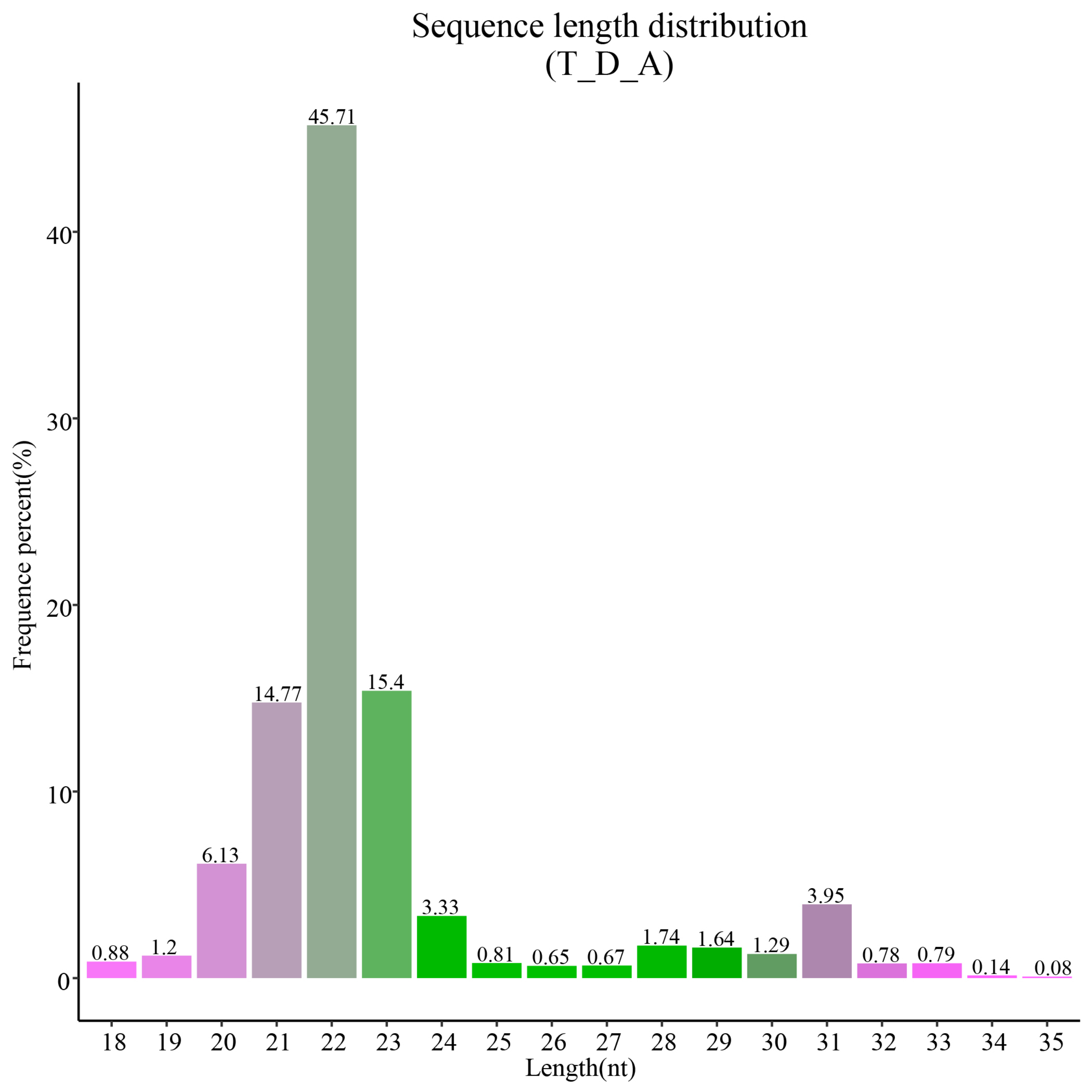
Sequence length distribution
(T_D_A)
Frequence percent(%)
0
10
20
30
40
45.71
14.77
15.4
6.13
3.33
3.95
0.88
1.2
0.81
0.65
0.67
1.74
1.64
1.29
0.78
0.79
0.14
0.08
18
19
20
21
22
23
24
25
26
27
28
29
30
31
32
33
34
35
Length(nt)

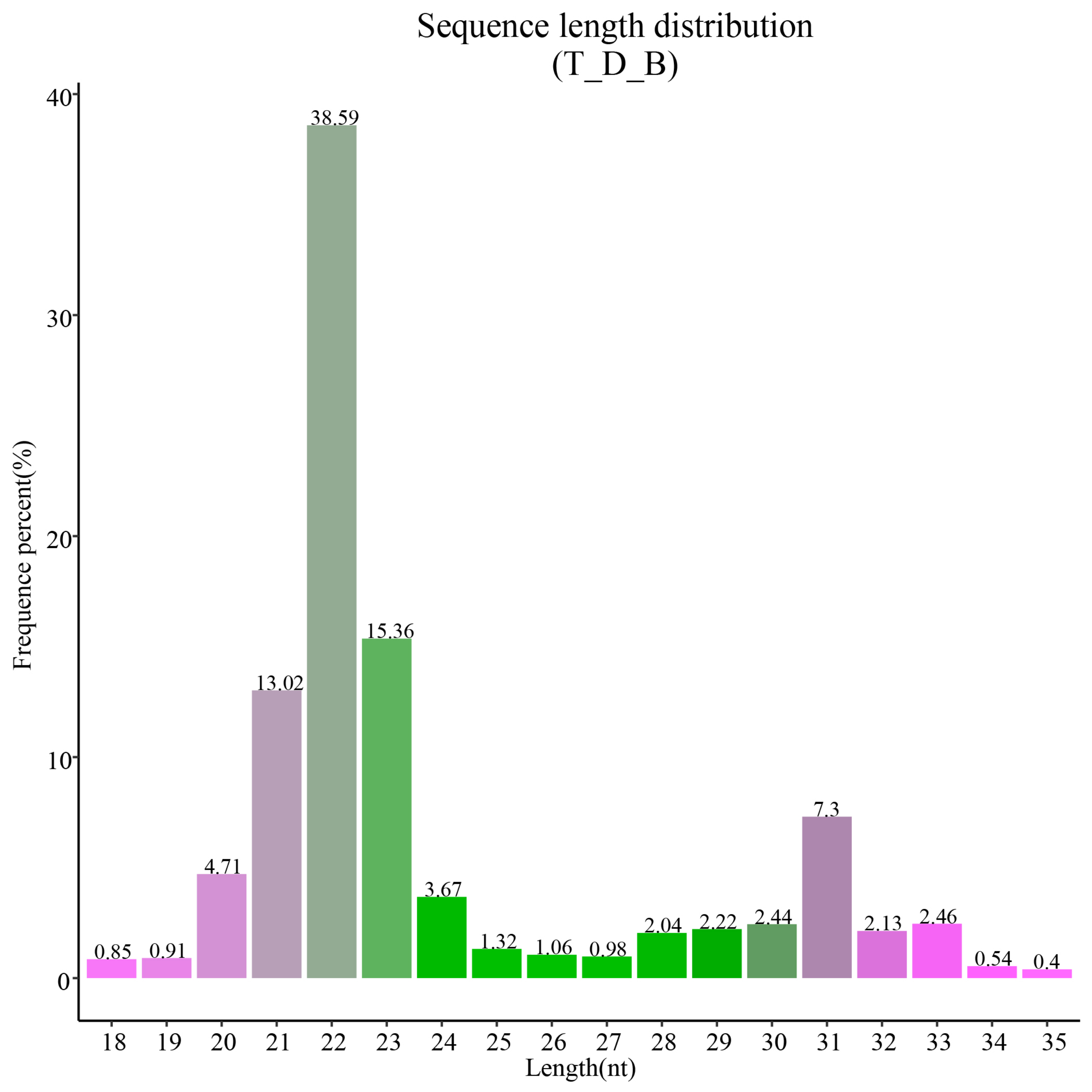

Sequence length distribution
(T_D_B)
Frequence percent(%)
40
30
20
10
0
38.59
15.36
13.02
7.3
4.71
3.67
1.32
1.06
0.98
2.04
2.22
2.44
2.13
2.46
0.85
0.91
0.54
0.4
18
19
20
21
22
23
24
25
26
27
28
29
30
31
32
33
34
35
Length(nt)
