# Supplementary material for: Screening and identification of miRNAs regulating Tbx4/5 genes of Pampus argenteus
Source: PeerJ. 2022 Oct 24;10:e14300. doi: 10.7717/peerj.14300 (PMC9610670; doi:10.7717/peerj.14300)
Supplement: Supplemental Information 3 — The horizontal axis represents the position of the miRNA bases, and the vertical axis represents the percentage of A/U/C/G bases in the miRNAs at the corresponding positions. [file peerj-10-14300-s003.pdf]

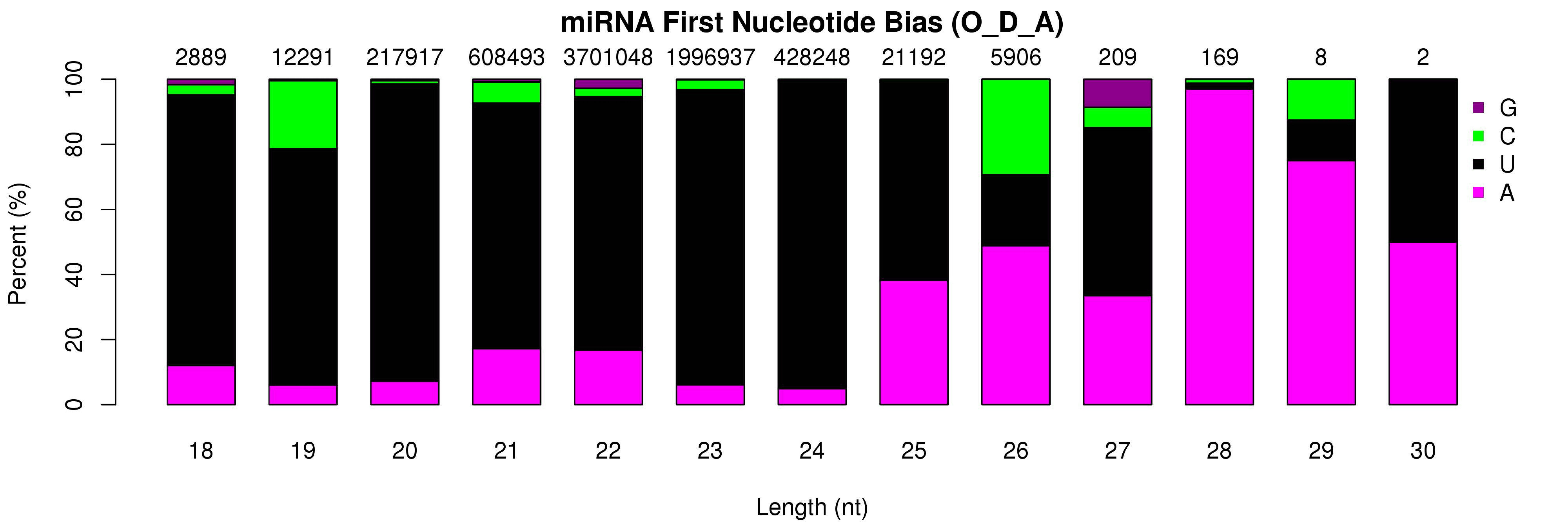

miRNA First Nucleotide Bias (O_D_A)
2889
12291
217917
608493
3701048
1996937
428248
21192
5906
209
169
8
2
Percent (%)
0
20
40
60
80
100
18
19
20
21
22
23
24
25
26
27
28
29
30
Length (nt)
G
C
U
A

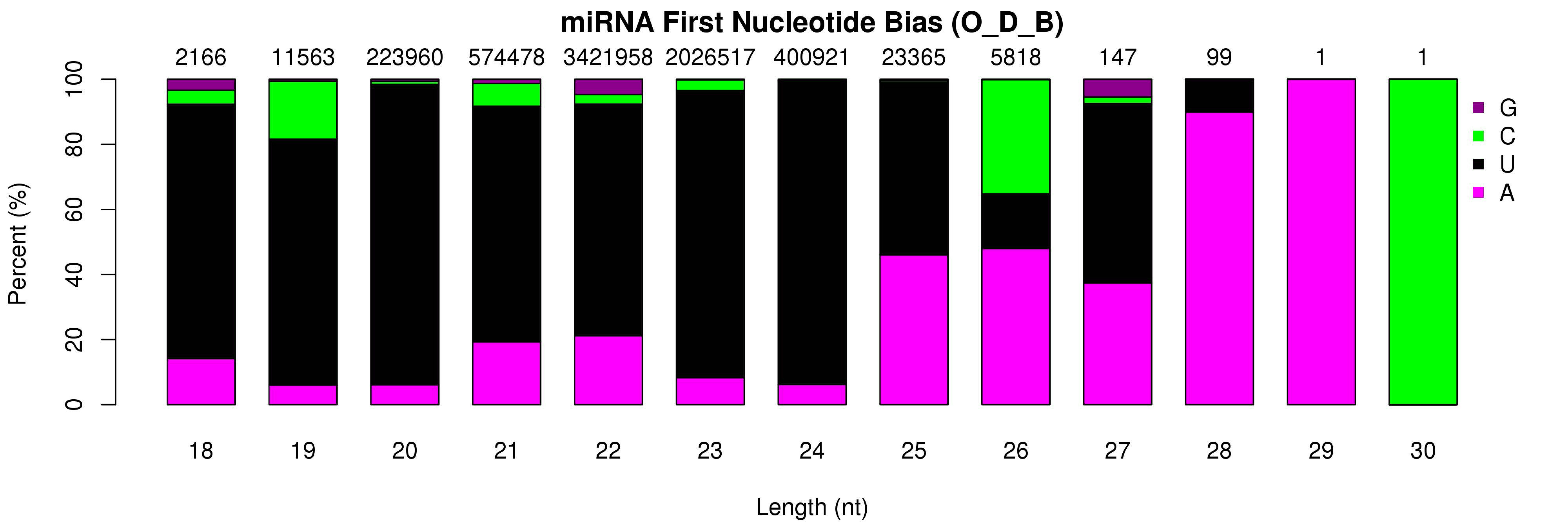

miRNA First Nucleotide Bias (O_D_B)
2166
11563
223960
574478
3421958
2026517
400921
23365
5818
147
99
1
1
Percent (%)
0
20
40
60
80
100
18
19
20
21
22
23
24
25
26
27
28
29
30
Length (nt)
G
C
U
A

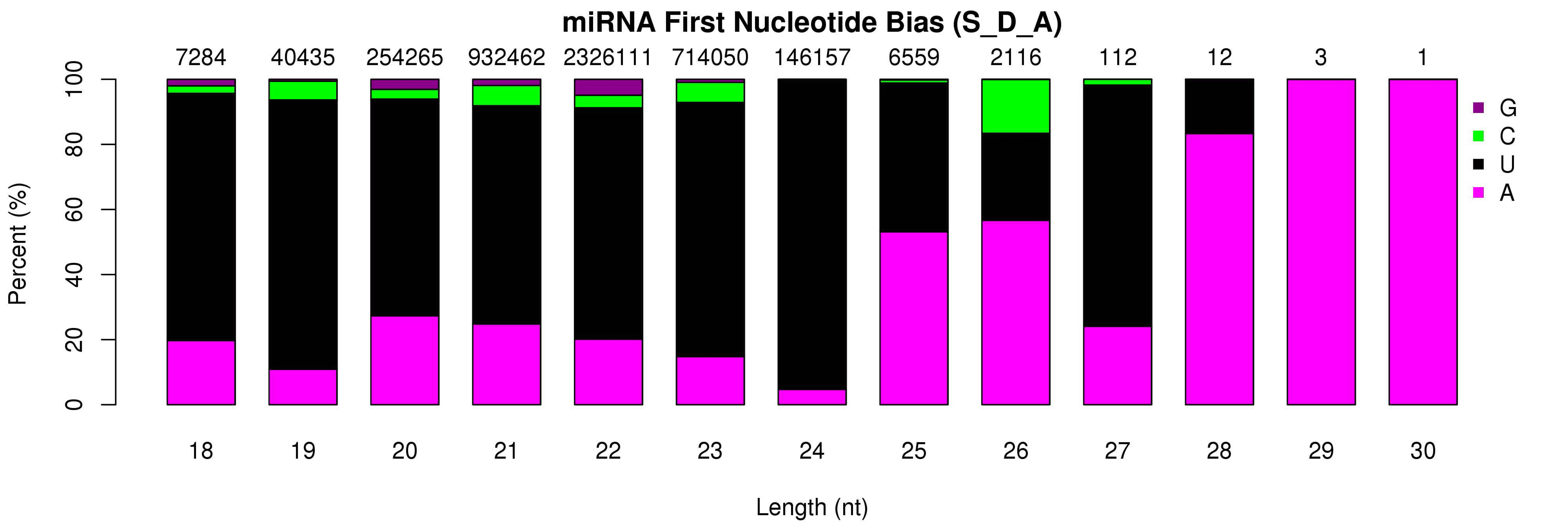
miRNA First Nucleotide Bias (S_D_A)
7284
40435
254265
932462
2326111
714050
146157
6559
2116
112
12
3
1
Percent (%)
0
20
40
60
80
100
18
19
20
21
22
23
24
25
26
27
28
29
30
Length (nt)
G
C
U
A

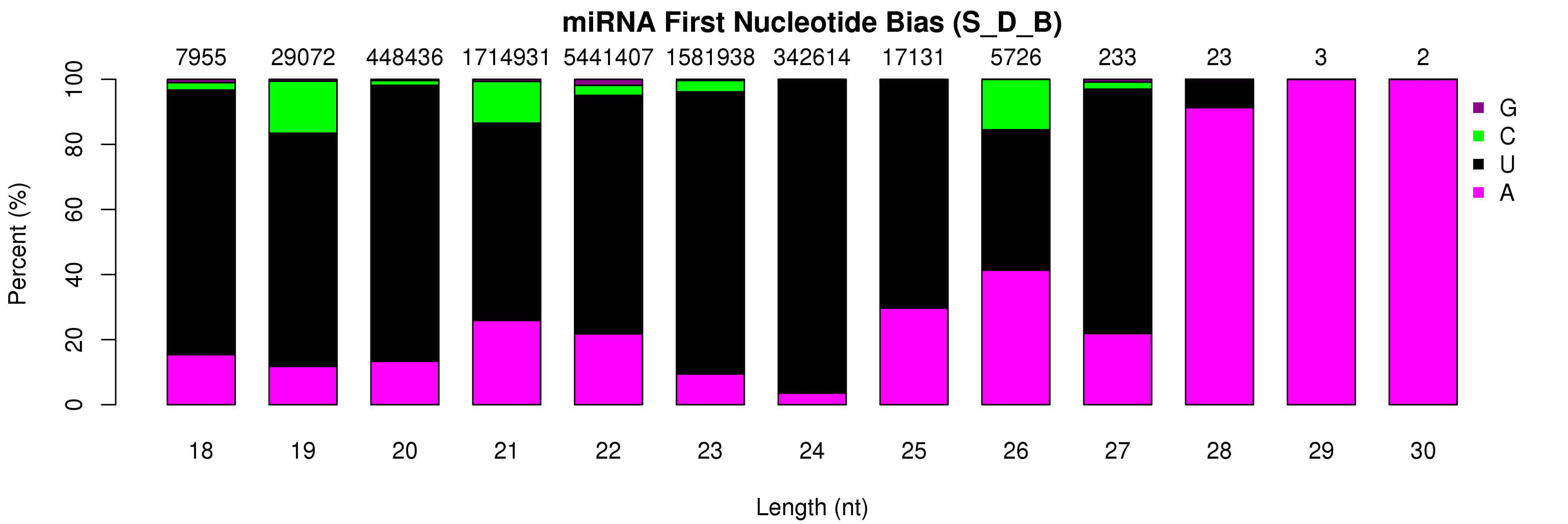
miRNA First Nucleotide Bias (S_D_B)
7955
29072
448436
1714931
5441407
1581938
342614
17131
5726
233
23
3
2
Percent (%)
0
20
40
60
80
100
G
C
U
A
18
19
20
21
22
23
24
25
26
27
28
29
30
Length (nt)

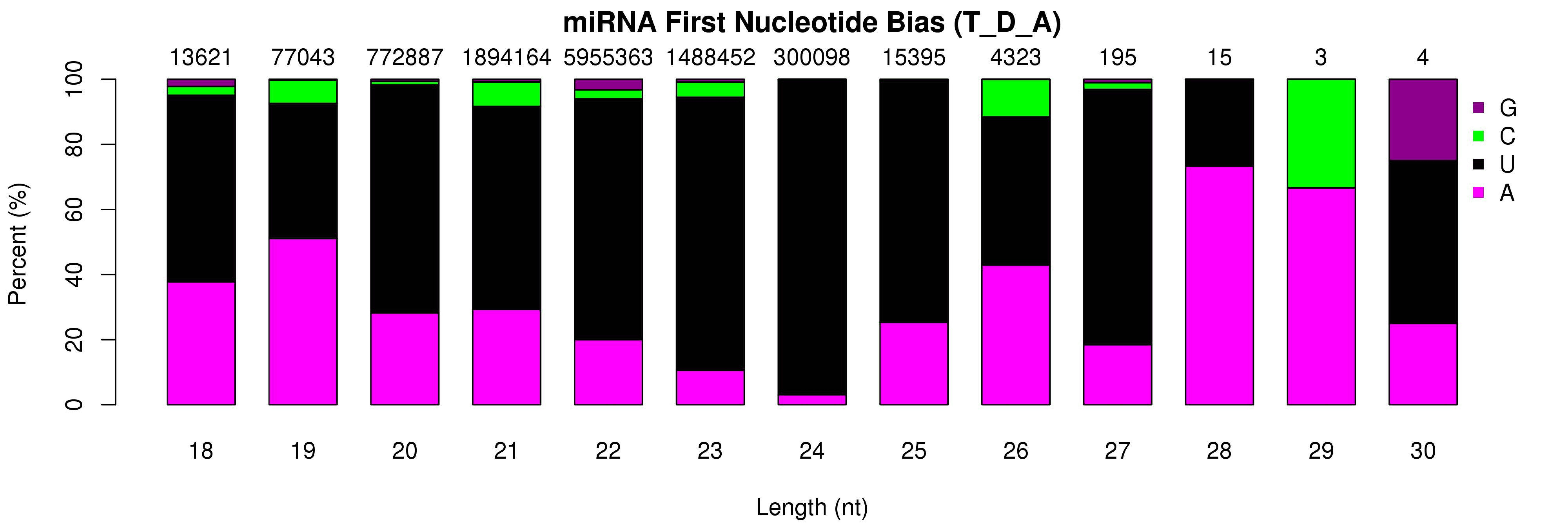
miRNA First Nucleotide Bias (T_D_A)
13621
77043
772887
1894164
5955363
1488452
300098
15395
4323
195
15
3
4
Percent (%)
100
80
60
40
20
0
G
C
U
A
18
19
20
21
22
23
24
25
26
27
28
29
30
Length (nt)

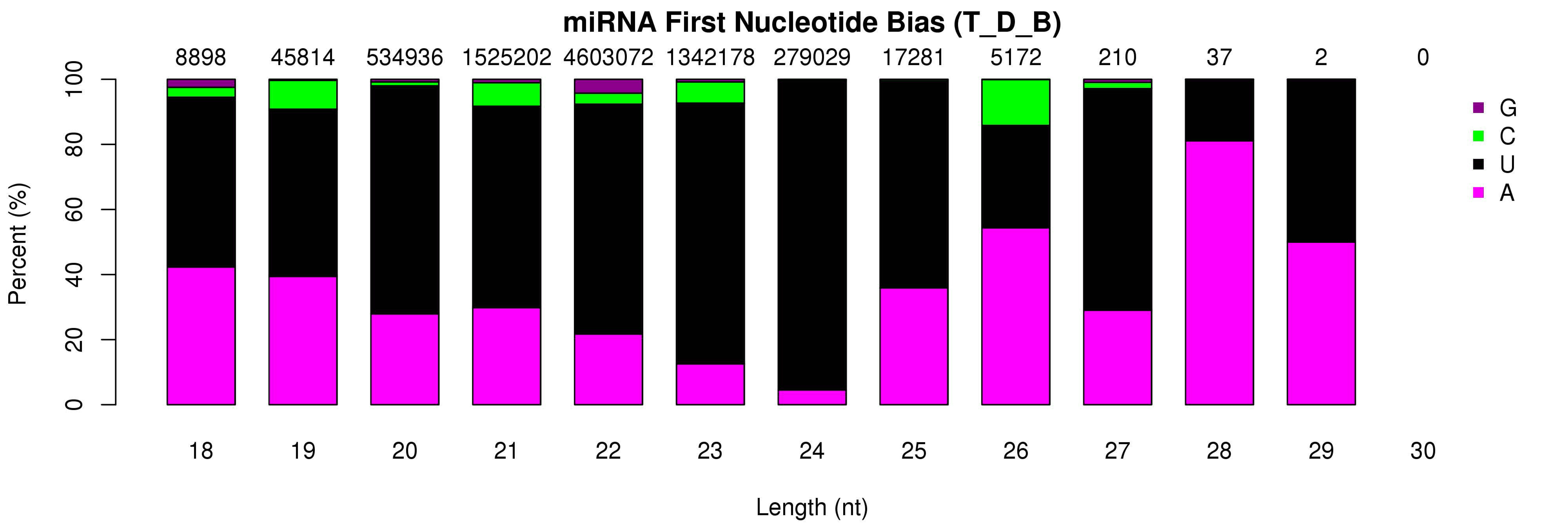

miRNA First Nucleotide Bias (T_D_B)
8898
45814
534936
1525202
4603072
1342178
279029
17281
5172
210
37
2
0
Percent (%)
0
20
40
60
80
100
18
19
20
21
22
23
24
25
26
27
28
29
30
Length (nt)
G
C
U
A
