# Supplementary material for: Screening and identification of miRNAs regulating Tbx4/5 genes of Pampus argenteus
Source: PeerJ. 2022 Oct 24;10:e14300. doi: 10.7717/peerj.14300 (PMC9610670; doi:10.7717/peerj.14300)
Supplement: Supplemental Information 4 — The horizontal axis represents the position of the miRNA bases, and the vertical axis represents the percentage of A/U/C/G bases in the miRNAs at the corresponding positions. [file peerj-10-14300-s004.pdf]

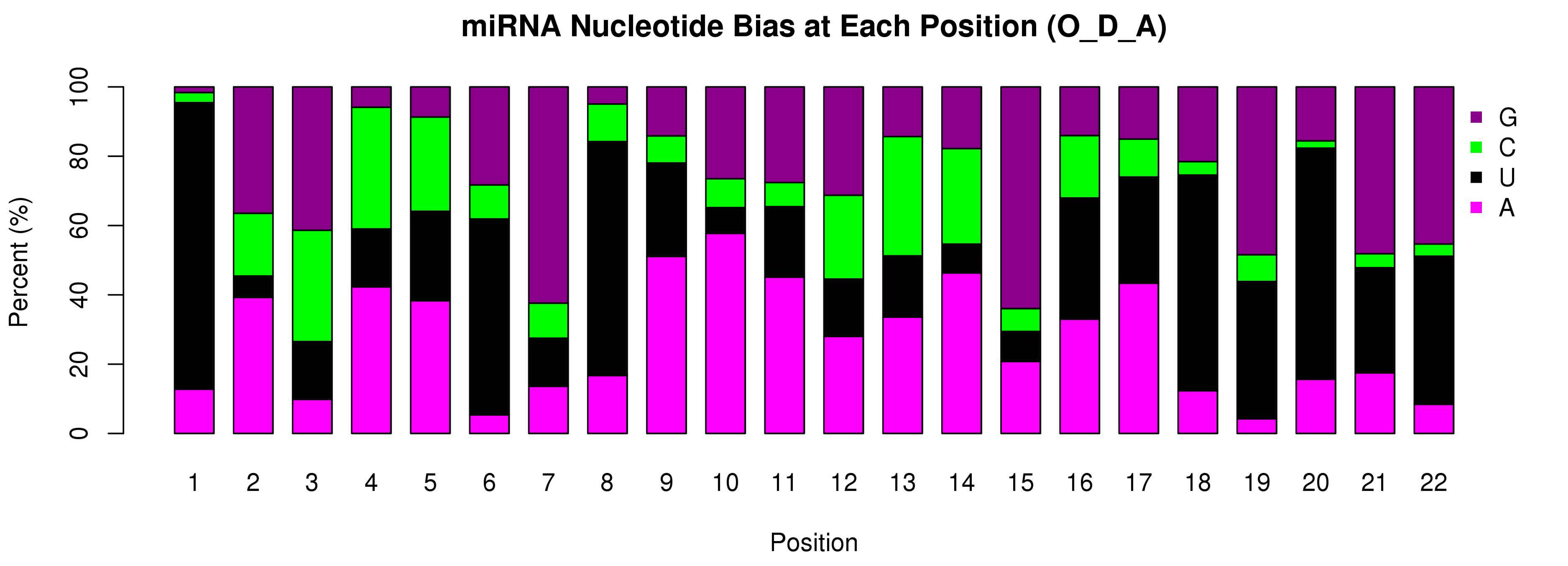

miRNA Nucleotide Bias at Each Position (O_D_A)
Percent (%)
0
20
40
60
80
100
1
2
3
4
5
6
7
8
9
10
11
12
13
14
15
16
17
18
19
20
21
22
Position
G
C
U
A

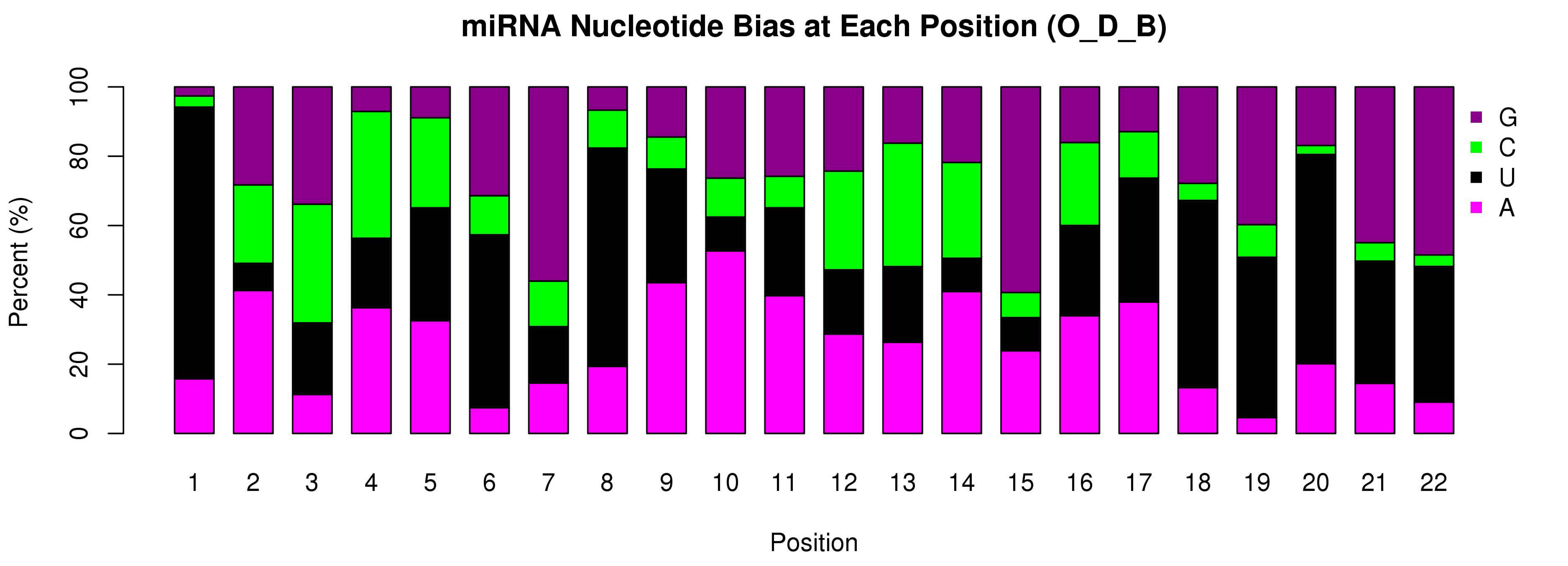
miRNA Nucleotide Bias at Each Position (O_D_B)
Percent (%)
0
20
40
60
80
100
1
2
3
4
5
6
7
8
9
10
11
12
13
14
15
16
17
18
19
20
21
22
Position
G
C
U
A

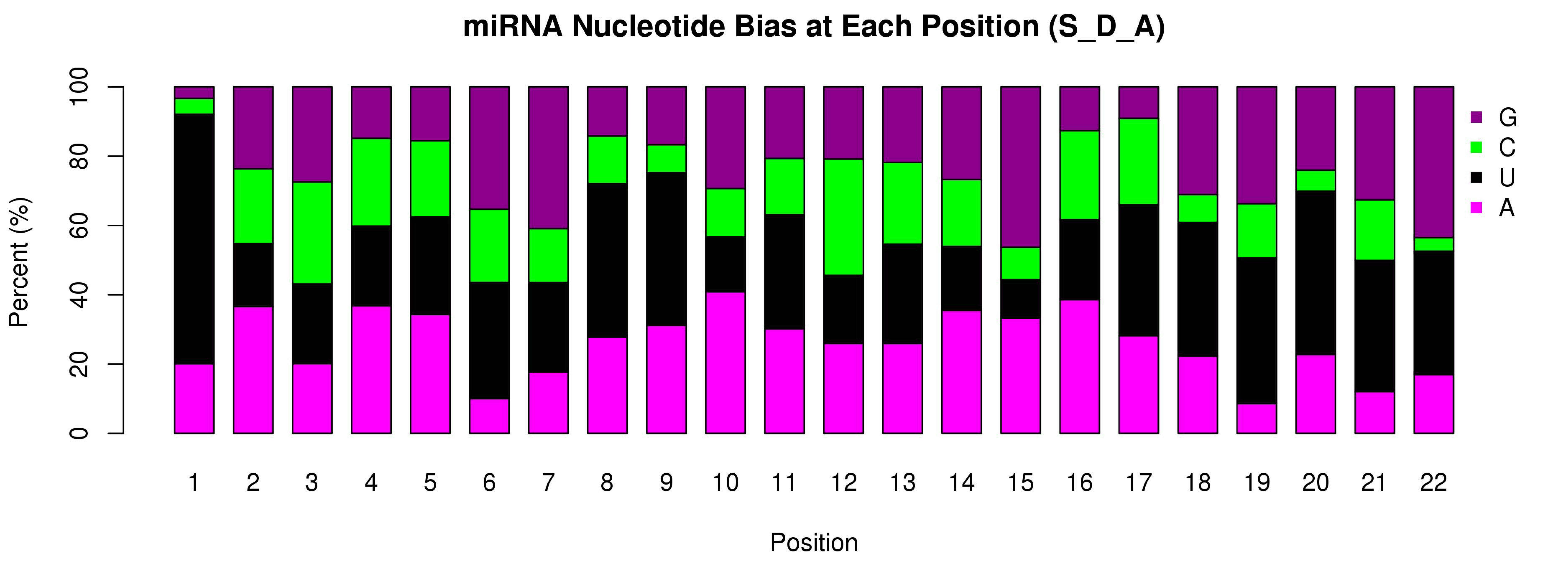

miRNA Nucleotide Bias at Each Position (S_D_A)
Percent (%)
0
20
40
60
80
100
1
2
3
4
5
6
7
8
9
10
11
12
13
14
15
16
17
18
19
20
21
22
Position
G
C
U
A

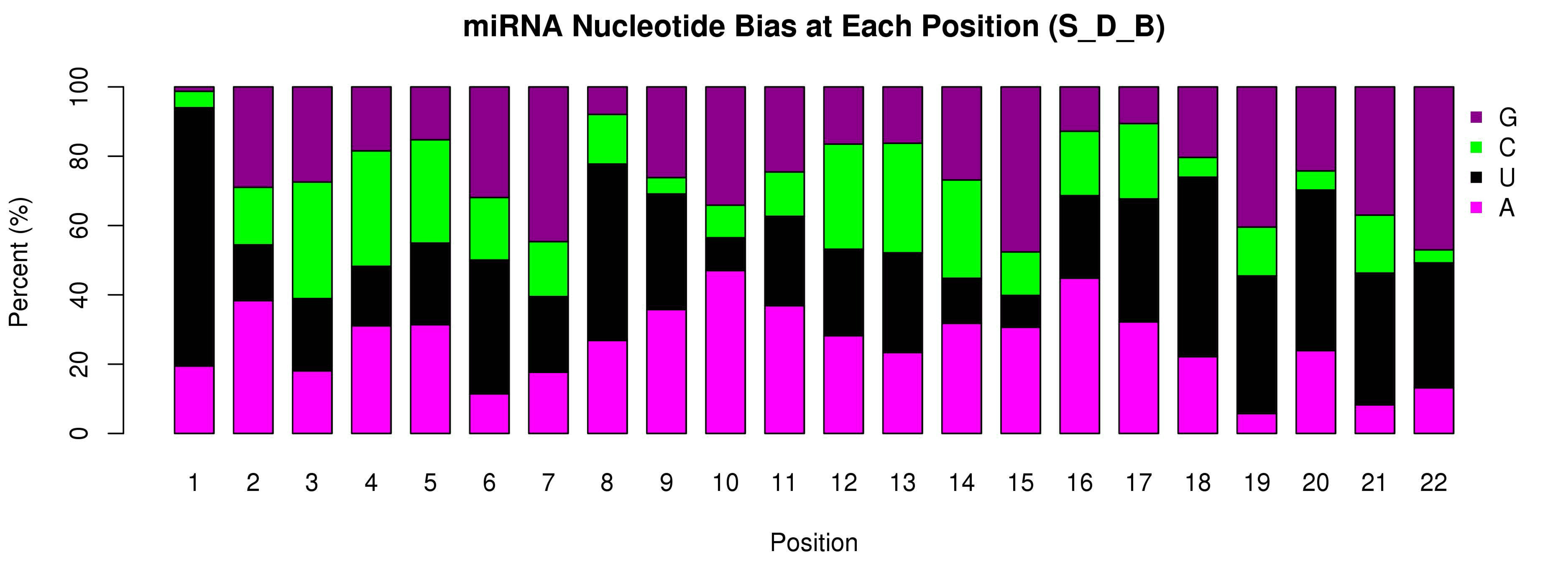
miRNA Nucleotide Bias at Each Position (S_D_B)
Percent (%)
0
20
40
60
80
100
1
2
3
4
5
6
7
8
9
10
11
12
13
14
15
16
17
18
19
20
21
22
Position
G
C
U
A

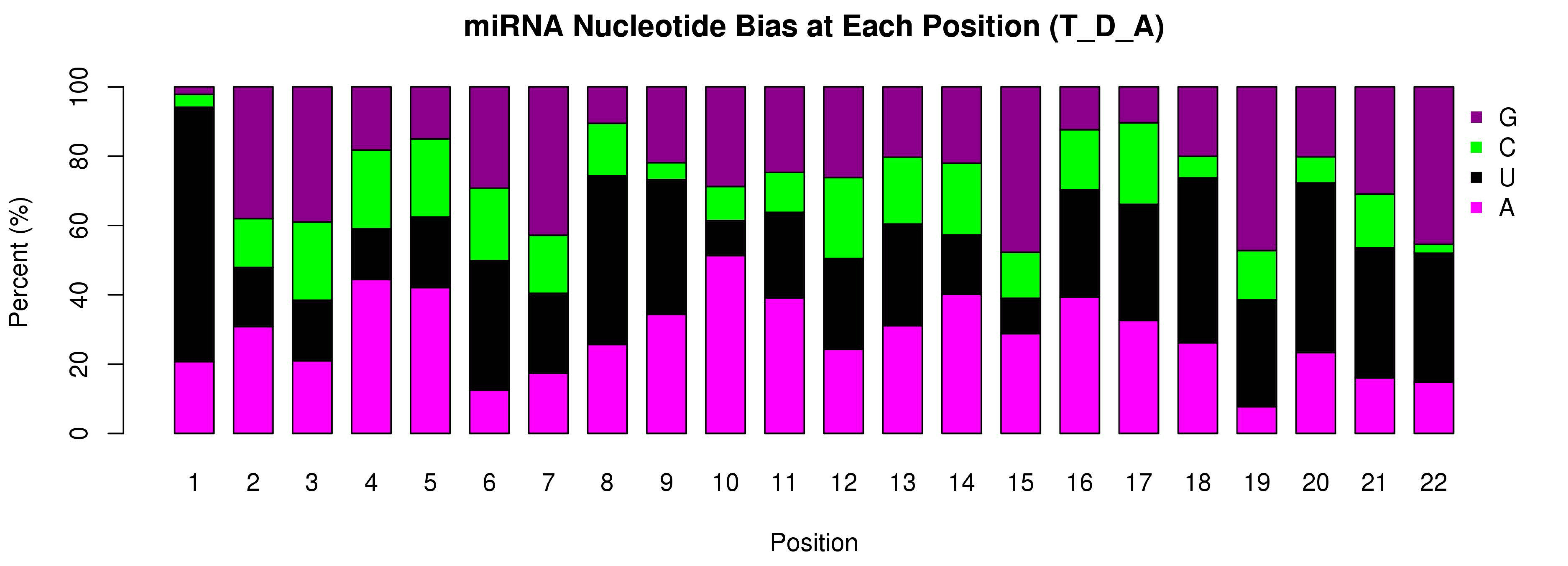
miRNA Nucleotide Bias at Each Position (T_D_A)
Percent (%)
0
20
40
60
80
100
1
2
3
4
5
6
7
8
9
10
11
12
13
14
15
16
17
18
19
20
21
22
Position
G
C
U
A

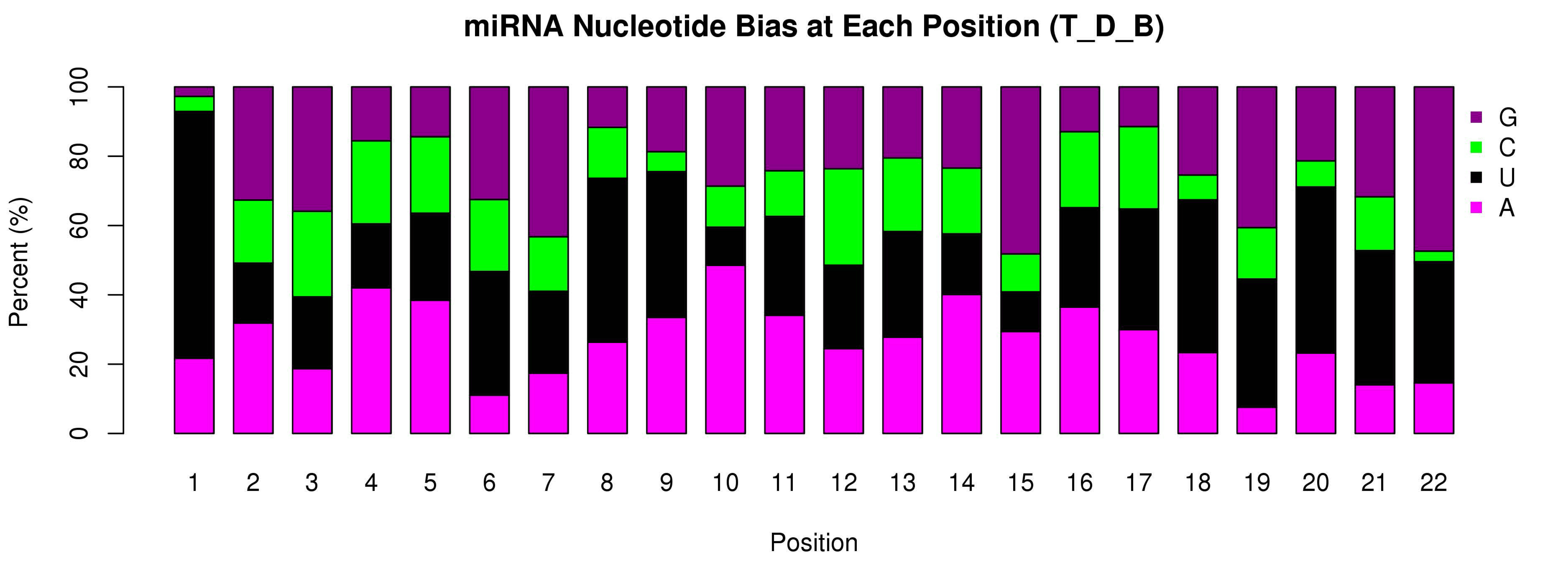

miRNA Nucleotide Bias at Each Position (T_D_B)
Percent (%)
0
20
40
60
80
100
1
2
3
4
5
6
7
8
9
10
11
12
13
14
15
16
17
18
19
20
21
22
Position
G
C
U
A
