# Supplementary material for: Screening and identification of miRNAs regulating Tbx4/5 genes of Pampus argenteus
Source: PeerJ. 2022 Oct 24;10:e14300. doi: 10.7717/peerj.14300 (PMC9610670; doi:10.7717/peerj.14300)
Supplement: Supplemental Information 5 — The double underlines at the beginning and tail regions represent the two restriction sites and protective bases in the 3′-UTR fragment. The labeled residues indicate the binding sites of the miRNA and sequences complementary to the 3′-UTR. [file peerj-10-14300-s005.pdf]

CTCGAGTTCTAGGCGATCGCTCGAGAGTACCAAGTGGGCCTGAGTAGTGCAGGGACTCAC
TGGACTGATAGCTAATGTGGGCCACTGGAGAAACCAGCTCTGTCCAACCTTCAAATCTGA
AATTAAACAAGCTGCAAACACCATGCAATGGTAATGAAATACAGCTATGATCATTACCCA
GTTGCACT(dre-miR-301c-3p)
CCAAAGCCATTTAAATCCATGAATTCTCACTGAAGCACAATTACGACAATGCTGTTTGTAGT
GAACAGTGGTTGACCTCCTCTTTACTTATGCAGAAGTGTGGCATATGTACTATAAGCCTGTG
ATTGGTTGGGTGGTTTTCTCATGTTCAAATGCAAAATATCAGAGGGAGCTTTAAGCTCAAGG
GTACATAGGTGTTACCACAATATGAGGTTTGACAGCTCACTCGCTAACAACTGTCTAAGTAG
TCTTAGCTAGCTCAGCTGTAGTAACAAGAAGGGTAACTTTTTGGATGGTAACAGACAAGGG
AAAATAAGCAACTTATGTGCTTGCTTGAAAATAATGTGAAAGCCATTCAGATATGTAGGATAT
GTGTACACGATGGTCACCAAGGGAAGCCAGGTACTGAAGGTTTCCCTGGGTGAGCATTTCT
TGCAGAGCTTCACATAGATAGAGCACATTTAATTAATGAAGCTGGTGTTAATTTACTTATTGT
AAACAAATCCCACGAACAGAAAAACAACAACAATGTGTATGTGTCTAAGTAACAGTGGAG
CTCTATGGCACATAGGAATAGGAAATATCAGTTTTGATACACAAACAGTGTTTGTTAGTAGG
ATCAGTTTATTGTTGGTTTAATCTTTTCATGGGATTTGTTGCCAGTAAAAAATAAATATTGGC
AACCTCAGCCTTTAAATCGGGTTTGATGCCCAAAAGTTACTAGATTTAAAGACATCACAACC
CAGATCCCACTTATTTGCATGTGCCAAAATGAGATT(novel_589)
TCTTACACCATGATGAATAAGGTTAAAGAGTGCATTTTATTAACAGAGGTTGGACATATC(novel_113)
AGAATATAGAGAAATTTAGGTTCTTGGCACATATATACTAAGAATCGTATGAGTAAAGACATA
CATTTGCTATGTGTATGACAGTCTTCCCATGGTGTACACACACAGAAATTAAATCAAATGAA
AGCTATGACACTCATGATAAGAGTTAACAATGCAGAGGTCTTGTCCATATACATTCTACAGG
TTATGAATCAGACTGGCCCATAATACTGTGGTTGATTGTCAAAG(dre-miR-301b-5p) (Thick line: novel_102)
CTTGGCTCCAAACTCACAACTCCAAACAACAATCTAGTGAAACAGGACAGGACAGTAAAG
AACTCTCAGGCAGAACCAGATGGGAGCTTAAAGAACTGTTGTTAAGTCATGGCGAGTACCG
GAAAAGAGAGCTGCTCACAAATCCCACTCCCAAATGACTGACATGGAAAAAACCCTTGCAAAAC (dre-miR-19b-5p)
TGCAAATGTAAAACAGCCTGAGTGGGCAGGGAAGTAGAAAGGAAAGGCTCTCAGTATCAT
TACTTACAATAGAGTTTGATGAACTTGCATTGATGAGGAAAGATTATTTGTAGGACAGGATG
ATGATCCCTTATGCATTTGTGCATTGCCCCTTATTTCATGTTGTTAAATTCAGTATAACATTTTG
TTTGAATATTTTGACATTTTTGATAAATAAGCGGCCGC
ACGTTTTGAGAAAGAGAAGAAAAAAAAAAAAAAAAAAAAA
