# Supplementary material for: Screening and identification of miRNAs regulating Tbx4/5 genes of Pampus argenteus
Source: PeerJ. 2022 Oct 24;10:e14300. doi: 10.7717/peerj.14300 (PMC9610670; doi:10.7717/peerj.14300)
Supplement: Supplemental Information 7 — The double underlines at the beginning and tail regions represent the two restriction sites and protective bases in the 3′-UTR fragment. The labeled residues indicate the binding sites of the miRNA and sequences complementary to the 3′-UTR. [file peerj-10-14300-s007.pdf]

CTCGAGTTCTAGGCGATCGCTCGAGAGTACCAAGTGGGCCTGAGTAGTGCAGGGACTCACT
GGACTGATAGCTAATGTGGGCCACTGGAGAAACCAGCTCTGTCCAACCTTCAAATCTGAAA
TTAAACAAGCTGCAAACACCATGCAATGGTAATGAAATACAGCTATGATCATTACCCAGTTG
CACT (dre-miR-301c-3p)
Mut:AATGCTG
CCAAAGCCATTTAAATCCATGAATTCTCACTGAAGCACAATTACGACAATGCTGTTTGTAGT
GAACAGTGGTTGACCTCCTCTTTACTTATGCAGAAGTGTGGCATATGTACTATAAGCCTGTG
ATTGGTTGGGTGGTTTTCTCATGTTCAAATGCAAAATATCAGAGGGAGCTTTAAGCTCAAGG
GTACATAGGTGTTACCACAATATGAGGTTTGACAGCTCACTCGCTAACAACTGTCTAAGTAG
TCTTAGCTAGCTCAGCTGTAGTAACAAGAAGGGTAACTTTTTGGATGGTAACAGACAAGGG
AAAATAAGCAACTTATGTGCTTGCTTGAAAATAATGTGAAAGCCATTCAGATATGTAGGATAT
GTGTACACGATGGTCACCAAGGGAAGCCAGGTACTGAAGGTTTCCCTGGGTGAGCATTTCT
TGCAGAGCTTCACATAGATAGAGCACATTTAATTAATGAAGCTGGTGTTAATTTACTTATTGT
AAACAAATCCCACGAACAGAAAAACAACAACAATGTGTATGTGTCTAAGTAACAGTGGAG
CTCTATGGCACATAGGAATAGGAAATATCAGTTTTGATACACAAACAGTGTTTGTTAGTAGG
ATCAGTTTATTGTTGGTTTAATCTTTTCATGGGATTTGTTGCCAGTAAAAAATAAATATTGGC
AACCTCAGCCTTTAAATCGGGTTTGATGCCCAAAAGTTACTAGATTTAAAGACATCACAACC
CAGATCCCACTTATTTGCATGTGCCAAAATGAGATT (novel_589)
novel_589 Mut: ACTCTAA
TCTTACACCATGATGAATAAGGTTAAAGAGTGCATTTTATTAACAGAGGTTGGACATATC
(novel_113) mut:GTGACAG
AGAATATAGAGAAATTTAGGTTCTTGGCACATATATACTAAGAATCGTATGAGTAAAGACATA
CATTTGCTATGTGTATGACAGTCTTCCCATGGTGTACACACACAGAAATTAAATCAAATGAA
AGCTATGACACTCATGATAAGAGTTAACAATGCAGAGGTCTTGTCCATATACATTCTACAGG
TTATGAATCAGACTGGCCCATAATACTGTGGTTGATTGTCAAAG (dre-miR-301b-5p) (Thick line: novel_102)
dre-miR-301b-5p mut: CAGTTTC novel_102 mut: AGCACTTC
CTTGGCTCCAAACTCACAACTCCAAACAACAATCTAGTGAAACAGGACAGGACAGTAAAG
AACTCTCAGGCAGAACCAGATGGGAGCTTAAAGAACTGTTGTTAAGTCATGGCGAGTACCG
GAAAAGAGAGCTGCTCACAAATCCCACTCCCAAATGACTGACATGGAAAAAACCCTTGCA
AAAC (dre-miR-19b-5p)
dre-miR-19b-5p Mut: ATGCTCGA
TGCAAATGTAAAACAGCCTGAGTGGGCAGGGAAGTAGAAAGGAAAGGCTCTCAGTATCAT
TACTTACAATAGAGTTTGATGAACTTGCATTGATGAGGAAAGATTATTTGTAGGACAGGATG
ATGATCCCTTATGCATTTGTGCATTGCCCCTTATTTCATGTTGTTAAATTCAGTATAACATTTTG
TTTGAATATTTTGACATTTTTGATAAATAAGCGGCCGC
ACGTTTTGAGAAAGAGAAGAAAAAAAAAAAAAAAAAAAAAA
